# Electrodeposited Manganese Dioxides and Their Composites as Electrocatalysts for Energy Conversion Reactions

**DOI:** 10.1002/cssc.202401907

**Published:** 2024-11-15

**Authors:** Masaharu Nakayama, Wataru Yoshida

**Affiliations:** ^1^ Department of Applied Chemistry Graduate School of Sciences and Technology for Innovation Yamaguchi University 2-16-1 Tokiwadai Ube 755-8611 Japan; ^2^ Blue Energy Center for SGE Technology (BEST) 2-16-1 Tokiwadai Ube 755-8611 Japan

**Keywords:** Manganese dioxide, Electrodeposition, Electrocatalysis, Energy conversion, Composites

## Abstract

Enhancing the efficiencies of electrochemical reactions for converting renewable energy into clean chemical fuels as well as generating clean energy is critical to achieving carbon neutrality. However, this enhancement can be achieved using materials that are not constrained by resource limitations and those that can be converted into devices in a scalable manner, preferably for industrial applications. This review explores the applications of electrochemically deposited manganese dioxides (MnO_2_) and their composites as electrochemical catalysts for oxygen evolution (OER) and hydrogen evolution reactions for converting renewable energy into chemical fuels. It also explores their applications as electrochemical catalysts for oxygen reduction reaction (ORR) and bifunctional OER/ORR for the efficient operation of fuel cells and metal–air batteries, respectively. Manganese is the second most abundant transition metal in the Earth′s crust, and electrodeposition represents a binder‐free and scalable technique for fabricating devices (electrodes). To propose an improved catalyst design, the studies on the electrodeposition mechanism of MnO_2_ as well as the fabrication techniques for MnO_2_‐based nanocomposites accumulated in the development of electrodes for supercapacitors are also included in this review.

## Graphical abstract

This review focuses on the electrochemical synthesis of MnO_2_ materials and their composites and their application as electrocatalysts for OER and HER reactions for the conversion of renewable energy into chemical fuels. The electrocatalytic function of electrodeposited MnO_2_ for ORR and bifunctional ORR/OER for efficient operation of fuel cells and metal‐air batteries is also reviewed.

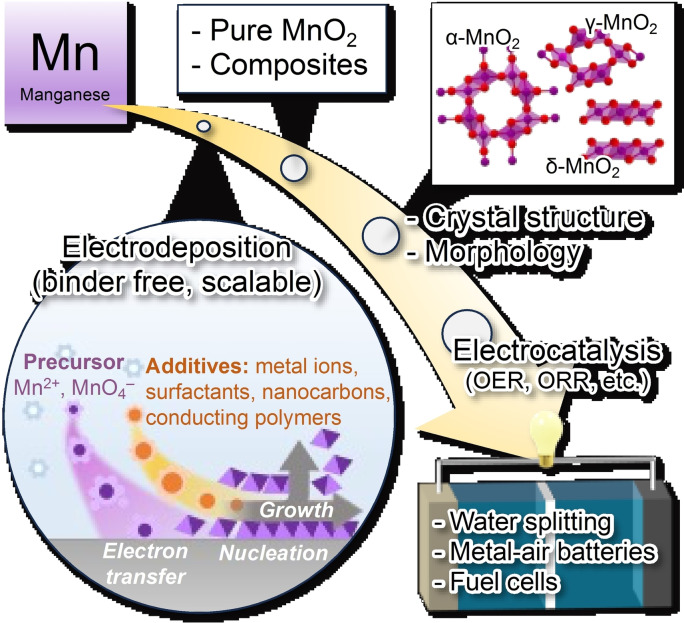

## 1 Introduction

The international community is confronted by dual challenges: the simultaneous tackling of environmental and energy crises as well as the pursuit of economic growth and development. Therefore, developing technologies for converting primary energy sources (e. g., solar and wind energies) into clean, high‐energy‐density chemical fuels (e. g., hydrogen) is essential. Such technologies include those deployed for hydrogen production (e. g., water splitting, metal–air batteries, and rechargeable fuel cells (FCs)). Additionally, these technologies are based on electrochemical reactions. Particularly, the efficiencies of the aforementioned systems are determined by the oxygen‐evolution reaction (OER) driven by water oxidation as well as the oxygen‐reduction reaction (ORR). Therefore, it is crucial to develop suitable electrocatalysts for such oxygen‐related reactions, and researchers worldwide are considerably committing to this.

It is widely acknowledged that iridium(IV)oxide (IrO_2_) and ruthenium(IV)oxide (RuO_2_) deliver optimal performances in OER, whereas Pt and Pd deliver superior performances in ORR.[^1^, ^2^] However, the scarcity and expensiveness of these materials present significant challenges that may intensify with the introduction of new technologies. To address these challenges, researchers have attempted to substitute precious metal–based catalysts with transition metal oxides. Among the transition metal oxides, manganese oxides have attracted considerable interest for a wide range of applications owing to their diverse valence states (from Mn^2+^ to Mn^7+^) as well as their crystalline structures, morphologies, and abundance.^[3]^ Following the introduction of zinc–manganese dioxide (MnO_2_) batteries by Leclanché in 1864, MnO_2_ has emerged as a raw material for fabricating alkaline batteries.^[4]^ Additionally, a highly efficient oxygen‐evolving catalyst is involved in photosystem II, which has a disordered chair structure comprising a manganese and calcium (Mn_4_CaO_5_) cluster.^[5]^ Inspired by the high OER efficiency of this biological system, numerous research groups have explored nanosized MnO_x_ and their derivatives.[^6^, ^7^] Notably, MnO_2_ is a naturally occurring substance present in a variety of crystalline structures; it can be synthesized by several methods, including electrodeposition.

This review explores electrochemically synthesized manganese dioxides and their applications as electrocatalysts for energy conversion. Electrochemically synthesized MnO_2_ thin films are advantageous to chemically synthesized MnO_2_ powders because they can be deployed as electrodes immediately after their synthesis (and post‐treatment). Put differently, the requirement of mixing the chemically synthesized catalyst with a polymer binder (and a conducting additive), followed by casting the resulting ink, can be omitted for electrochemically synthesized MnO_2_ thin films. Further, the absence of binders eliminates the factors that impede the contact between the electrode and an electrolyte as well as the transfer of ions and electrons between them. Therefore, the selection and design of the electrode substrate, i. e., the scaffolds upon which the MnO_2_ or their composites are deposited, constitute a key research area. Numerous studies have extensively considered the development of MnO_2_‐based supercapacitor electrodes.[^8^, ^9^, ^10^] MnO_2_ has been deployed in numerous applications, including electrochemical energy storage (EES) devices, such as primary alkaline batteries, rechargeable batteries, supercapacitors, and recently, zinc‐ion batteries (ZIBs), as well as catalysts. Consequently, comprehensive reviews have been published for each application. Nevertheless, only a few reviews have been based on electrodeposited MnO_2_ and their composites, providing in‐depth analyses of their syntheses and electrocatalysis. In the field of electrochemistry, recently published reviews on manganese oxides were mainly focused on the electrode materials for supercapacitors[^11^, ^12^, ^13^, ^14^, ^15^] and Mn‐based cathodes for rechargeable aqueous batteries.[^16^, ^17^, ^18^, ^19^] Additionally, water‐oxidizing catalysis using MnO_2_‐based nano/micro materials, which are not necessarily electrochemically synthesized, was the focus of those published reviews.^[20]^ Furthermore, the environmental applications of MnO_2_‐based materials have been reviewed.[^21^, ^22^] To the best of our knowledge, however, the electrochemical synthesis of MnO_2_ materials and their composites, which can function as catalysts for energy conversion reactions, has not yet been summarized.

In each application area, the extant studies mainly explored single‐component MnO_2_. Subsequently, composite materials incorporating other metallic elements, nanocarbons, and conducting polymers were developed to enhance MnO_2_ performance. The auxiliary materials can be co‐deposited with MnO_2_ or synthesized chemically or electrochemically before or after MnO_2_ electrodeposition. Furthermore, the scaffolds for MnO_2_ deposition (electrode substrates), including various three‐dimensional (3D) porous materials (e. g., nanostructured carbons and metal foams), play significant roles in enhancing the performances of MnO_2_ electrodes.

Notably, this review employs the term, “electrolytic manganese dioxide (EMD),” in a more restricted sense. EMDs have long been employed in the alkaline‐battery industry; they are produced as massive powders via the anodic electrolysis of strongly acidic aqueous solutions of manganese(II) sulfate (MnSO_4_) + sulfuric acid (H_2_SO_4_; 0.1–5.0 M) at high temperatures (>80 °C), exhibiting a γ‐type crystalline structure.^[11]^ Conversely, the term, “electrodeposited manganese dioxides,” as employed in this review encompasses a more expansive range of materials. These materials are formed from aqueous solutions containing precursor Mn species (Mn^2+^ and MnO_4_^−^) or dispersed MnO_2_ colloidal particles on various conductive substrates via various electrochemical operations and exist mainly as thin films.

In Section 2, we offer a concise overview of the thermodynamic properties (Section 2.1) as well as crystalline polymorphs (Section 2.2) of pure MnO_2_. Section 3 addresses the various electrodeposition modes for MnO_2_ based on the fundamental principles of materials fabrication discussed in Section 4. Section 4 further offers an overview of the fabrication of various nanocomposites, many of which were synthesized for supercapacitor electrodes. However, the information presented will sufficiently benefit the electrocatalyst synthesis, which is the focus of this review. Nevertheless, this review avoids offering an exhaustive account of the performance of these materials as supercapacitor electrodes. Section 5 furthers the discussion on the function of single‐component MnO_2_ and its composites, which had been synthesized by electrodeposition and deployed as electrocatalysts. The reactions include the water‐splitting reaction (OER and hydrogen evolution reaction (HER)), which is a critical process of converting renewable‐energy sources into high‐energy‐density chemical fuels, and ORR, which is a pivotal reaction in FCs. Furthermore, the discussion will cover bifunctional catalysts for OER and ORR, which are essential for the reversible operations of metal–air batteries. Section 6 summarizes the review, highlighting the prospects.

## 2 Manganese Dioxides

MnO_2_ is a non‐toxic, black powder existing as a pyrolusite mineral in the Earth′s crust. MnO_2_ represents the principal form of manganese. It is primarily deployed as a cathode material in the alkaline‐battery industry, accounting for an annual demand of over 230,000 tons and an annual growth rate of over 9.6 %.^[11]^ EMDs, also deployed as cathode materials in alkaline primary batteries and lithium–manganese primary batteries, are among the most extensively deployed cathode materials in alkaline batteries. MnO_2_ plays a crucial role as a functional material in EES systems, such as supercapacitors and batteries, owing to its high specific capacity (1,370 F g^−1^ and 308 mA g^−1^) that is attributable to the redox capability of Mn ions.^[15]^ Furthermore, Mn oxides are advantageous owing to their abundance (accounting for the second most abundant transition metal near the Earth′s surface), low production costs, and environmental compatibility.

### 2.1 Thermodynamics

**Figure 1:**
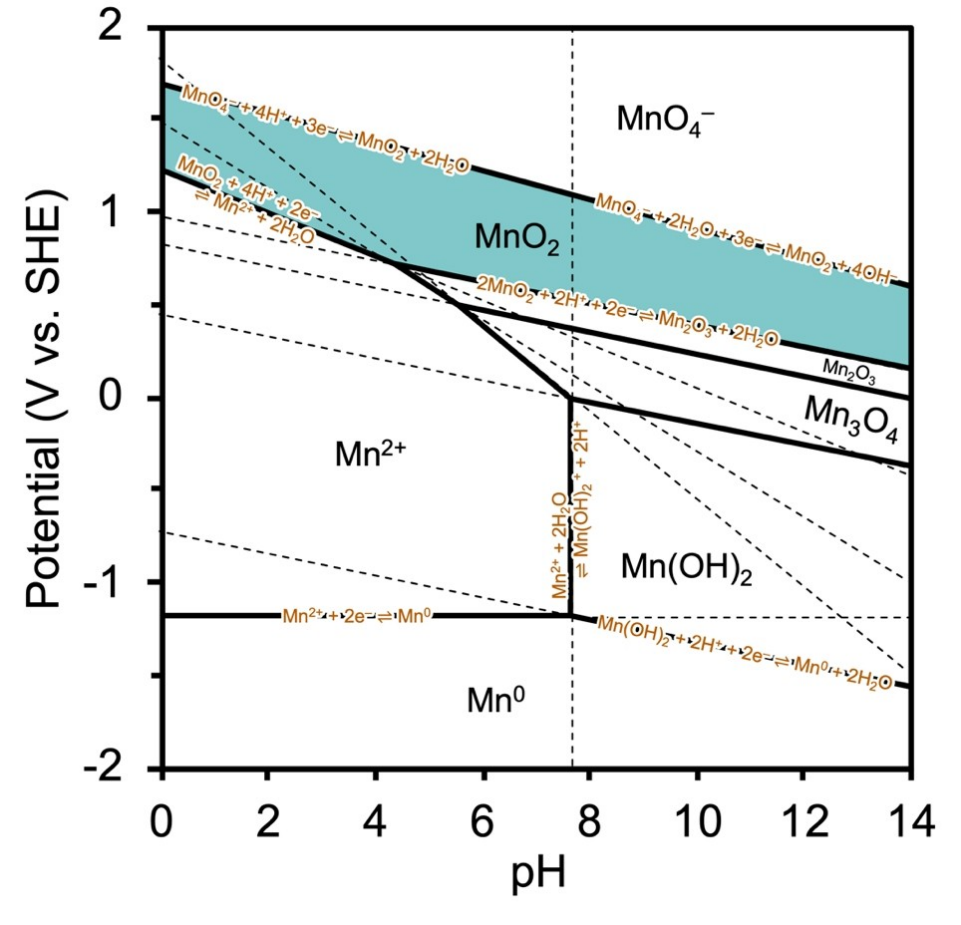
Pourbaix diagram of the Mn–H_2_O system (25 °C).

The various oxidation states of Mn account for the existence of diverse Mn oxides. The thermodynamic stability of these Mn‐based species depends on pH and potential, which can be effectively visualized using the Pourbaix diagram (Figure 1). Table 1 presents the redox‐equilibrium data of the Mn−H_2_O system along with their corresponding standard‐electrode potentials.^[23]^ Further, Figure 1 shows the regions in which the Mn atom, ions, and oxides are stable under varying pH and electrode‐potential conditions. For example, at low pH and potential values, Mn^2+^ is the dominant species, which can be converted into MnO_2_ if the potential is shifted to a more positive value. This is the fundamental principle underlying the electrochemical synthesis of MnO_2_. Conversely, the dissolved species, namely permanganate ions (MnO_4_^−^), exist at positive potentials over a wide pH range, and they are converted into MnO_2_ in the nearby negative potential region. Put differently, MnO_2_ can be synthesized via the anodic oxidation and cathodic reduction of Mn^2+^ and MnO_4_^−^, respectively (Figure 1), as will be comprehensively discussed in Section 3. Furthermore, it is well known that MnO_2_ is transformed into other solid‐phase oxides near neutral conditions, exhibiting lower oxidation states. Therefore, a comprehensive understanding of the thermodynamic principles governing the Mn−H_2_O system is essential to exploring the synthesis and application of Mn oxides. Notably, these thermodynamic aspects are closely linked to kinetic requirements, such as overpotentials, in electrochemistry and the presence of water and hydrogen redox equilibria, particularly in the context of employing Mn oxide catalysts in water splitting and other oxygen‐related reactions (Section 5).

**Table 1:** Redox equilibrium equations and potentials for the MnO_2_–H_2_O system.

Reaction	Potential
Mn^0^ ⇌ Mn^2+^+2e^−^	*E*=−1.18+0.03 log $a_{{Mn}^{2+}}$
Mn^2+^+2H_2_O ⇌ Mn(OH)_2_+2H^+^	pH=−7.65−0.5 log $a_{{Mn}^{2+}}$
3Mn^2+^+4H_2_O ⇌ Mn_3_O_4_+8H^+^+2e^−^	*E*=1.81−0.09 log $a_{{Mn}^{2+}}$ −0.236pH
2Mn^2+^+3H_2_O ⇌ Mn_2_O_3_+6H^+^+2e^−^	*E*=1.49−0.06 log $a_{{Mn}^{2+}}$ −0.177pH
Mn^2+^+2H_2_O ⇌ MnO_2_+4H^+^+2e^−^	*E*=1.23−0.03 log $a_{{Mn}^{2+}}$ −0.118pH
MnO_2_+2H_2_O ⇌ MnO_4_^−^+4H^+^+3e^−^	*E*=1.70−0.079pH
Mn^0^+2H_2_O ⇌ Mn(OH)_2_+2H^+^+2e^−^	*E*=−0.729−0.059pH
3Mn(OH)_2_ ⇌ Mn_3_O_4_+2H_2_O+2H^+^+2e^−^	*E*=0.454−0.059pH
2Mn_3_O_4_+H_2_O ⇌ 3Mn_2_O_3_+2H^+^+2e^−^	*E*=0.830−0.059pH
Mn_2_O_3_+H_2_O ⇌ 2MnO_2_+2H^+^+2e^−^	*E*=0.974−0.059pH

### 2.2 Crystalline Structures

**Figure 2:**
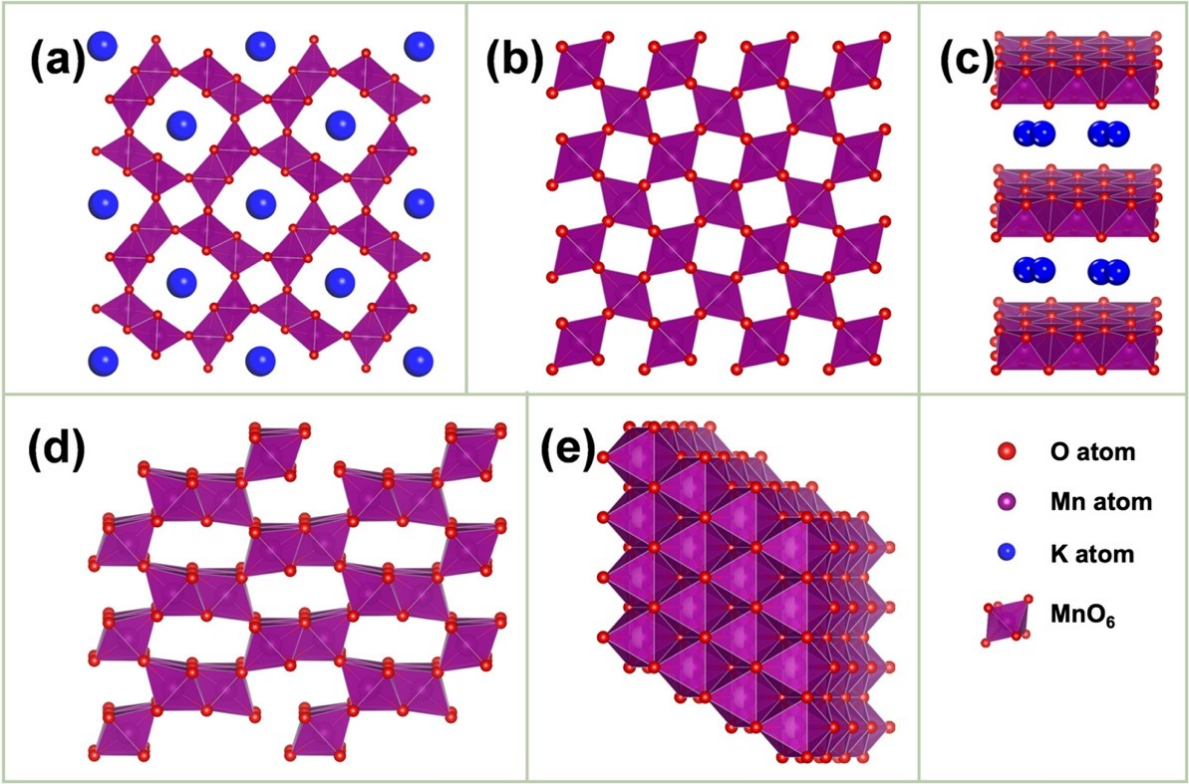
Crystalline polymorphs of the MnO_2_ composites: (a) α‐MnO_2_, (b) β‐MnO_2_, (c) δ‐MnO_2_, (d) γ‐MnO_2_, and (e) ε‐MnO_2_. Reproduced with permission from Ref.^[24]^ Copyright 2022, American Chemical Society.

Figure 2 shows that MnO_2_ assumes a variety of crystalline structures (α‐, β‐, γ‐, δ‐, ε−) with nanoscale stabilization.^[3]^ The diverse ranges of crystalline structures observed in MnO_2_ are attributable to the sharing of corners and edges between octahedral [MnO_6_] building blocks. In the [MnO_6_] unit, the central position is occupied by Mn^4+^, whereas six O^2−^ ions are located at the corners. The tunnel structures are also present in three dioxides, namely α‐, β‐, and γ‐MnO_2_, whereas δ‐MnO_2_ exhibits a layered structure. ε‐MnO_2_ consists of a hexagonally close‐packed oxygen framework where the Mn^4+^ are distributed randomly in one half of the octahedral sites. α‐MnO_2_ (hollandite) exhibits one‐dimensional (1D; 2×2) large tunnels (4.6×4.6 Å), which facilitate substantial storage as well as the rapid diffusion of foreign cations along the z‐axis. Notably, β‐MnO_2_ (pyrolusite) is among the most stable MnO_2_ compounds, exhibiting narrow (1×1) tunnels (2.3×2.3 Å). These narrow tunnels only accommodate small ions, such as H^+^ and Li^+^, and do not promote ion diffusion. The γ‐MnO_2_ phase exhibits an intergrowth tunnel structure comprising the pyrolusite (1×1) tunnels (2.3×2.3 Å) and ramsdellite (2×1) tunnels (4.6×2.3 Å). Additionally, δ‐MnO_2_ is designated birnessite; it is characterized by 1×∞ two‐dimensional (2D) channels with an interlayer distance of approximately 7 Å, which can accommodate metal cations and coordinated H_2_O molecules. As γ‐MnO_2_ undergoes a more complex growth process, it is less crystalline and exhibits a greater prevalence of defects and vacancies compared with other polymorphs. Among the various crystalline forms of MnO_2_, γ‐MnO_2_ (EMD) is the most commonly utilized electrode material in batteries as well as for other energy‐storage applications.^[11]^

## 3 Electrodeposition

The development of methods (based on various techniques, such as hydrothermal, sol–gel, microwave‐assisted, electrostatic‐spinning, template‐assisted, and electrodeposition methods) for synthesizing MnO_2_ with different morphologies, crystalline structures, porosities, and specific surface areas has facilitated the deployment of MnO_2_‐based materials. Hydrothermal synthesis was conducted in a Teflon‐lined autoclave, maintaining the aqueous precursor solution at a temperature of >100 °C.^[25]^ Sol‐gel synthesis of metal oxides can be performed via the hydrolysis and condensation of metal cations in aqueous solutions.^[26]^ Microwave‐assisted synthesis is a form of heating that employs microwave radiation to accelerate chemical reactions.^[27]^ The electrostatic spinning method involves the use of an electrostatic field created between electrodes (nozzle and collector) under the influence of high voltage to stretch a droplet of the precursor solution into a fiber.^[28]^

Among them, scholars have deployed electrodeposition for the straightforward production of diverse, flexible structures. Electrodeposition is a distinctive technique for fabricating metal oxides, polymers, and composite electrodes, as it allows for the straightforward modification of material properties, including material compositions, morphologies, and crystalline structures, through the manipulation of electrochemical variables. Additionally, this method is preferred in developing commercial energy conversion and storage systems as well as electrolyzers owing to its high purity, high MnO_2_ content, processing ease, and inherent scalable flexibility. The high purity of the product is achieved by the fact that the electrode‐supported product can be easily separated from the liquid phase containing the reactants. In fact, Gong *et al*. demonstrated that the MnO_2_ deposited galvanostatically on stainless steel 304 from MnSO_4_+(NH_4_)_2_SO_4_ contained no impurities, using EDAX and XPS depth profiling.^[29]^

The formation of material via electrodeposition onto a substrate is achieved via Faradaic or non‐Faradaic processes. As will be described in more detail, the former involves the deposition of soluble‐precursor Mn species (Mn^2+^ and MnO_4_^−^) onto the electrode substrate as insoluble MnO_2_ and its nonstoichiometric oxides (MnO_2–x_) via electron transport from the circuit. Conversely, the latter involves the dispersion of colloidal MnO_2_ nanoparticles (NPs), which have been synthesized at another site, in water or alcohol, followed by the application of an electric field between the two electrodes. This facilitates the electrophoretic migration of the charged particles toward the electrode with the opposite charge. Generally, “electrodeposition” refers to the former (Faradaic processes), unless stated otherwise. As outlined in Table 2, variations in the conditions for electrodeposition result in the formation of distinct crystal structures.

**Table 2:** Conditions for electrodepositions resulting in various crystalline structures of MnO_2_.

Crystalline structure	ED Bath composition	Anodic or Cathodic	ED Procedure	Post‐treatment	Ref.
γ‐MnO_2_	5 mM MnSO_4_+0.1 M H_2_SO_4_, 25 °C	Anodic	Potential cycling (0–1.5 V vs. Ag/AgCl)		[30]
δ‐MnO_2_	5 mM MnSO_4_+0.1 M Na_2_SO_4_, 25 °C	Anodic	Potential cycling (0–1.5 V vs. Ag/AgCl)		[30]
δ‐MnO_2_	2 mM MnSO_4_+50 mM tetraalkyl ammonium chloride, 25 °C	Anodic	Constant potential (1.0 V vs. Ag/AgCl)		[31]
δ‐MnO_2_	4 mM MnSO_4_+0.4 M Na_2_SO_4_, 25 °C	Anodic	Constant potential (1.0 V vs. Ag/AgCl)		[32]
ε‐MnO_2_	0.3 M MnSO_4_ (pH 7), 70 °C	Anodic	Constant current (100 mA cm^−2^)		[33]
Defective rock salt MnO_2_	0.3 M MnSO_4_+0.2 M EDTA (pH 7), 70 °C	Anodic	Constant current (100 mA cm^−2^)		[33]
Defective antifluorite MnO_2_	0.3 M MnSO_4_+0.3 M Na_3_C_6_H_5_O_7_, 70 °C	Anodic	Constant current (100 mA cm^−2^)		[33]
α‐MnO_2_	0.005 M Mn(NO_3_), 10°C	Cathodic	Constant current (−1 mA cm^−2^)	Annealing at 300°C	[34]
δ‐MnO_2_	0.02 M KMnO_4_, 25 °C	Cathodic	Constant current (−2 mA cm^−2^)		[35]
δ‐MnO_2_	0.002 M KMnO_4_+0.05 M KCl, 25 °C	Cathodic	Constant potential (0 V vs. Ag/AgCl)		[36]

### 3.1 Anodic Deposition

The formation of MnO_2_ via the two‐electron oxidation of Mn^2+^ is represented by the following general formula:
(1)

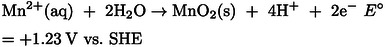




Paul *et al*.^[37]^ first explored the formation mechanism, after which Rodrigues *et al*.^[38]^ and Donne *et al*.[^39^, ^40^, ^41^] conducted further investigations. Their efforts were aimed at gaining insight into the EMD‐deposition mechanism using rotating ring‐disc electrode voltammetry and electrochemical quartz crystal microbalance (EQCM). Consequently, numerous mechanistic studies have been conducted in strongly acidic to acidic solutions comprising MnSO_4_ and H_2_SO_4_, clarifying that the amorphous hydrated‐form, low‐crystalline γ‐MnO_2_ are produced in acidic electrolytes, whereas δ‐MnO_2_ is formed under neutral to alkaline conditions.^[30]^ The following subsubsections discuss the electrodeposition mechanisms in acidic and neutral to alkaline electrolytes.

#### 3.1.1 Acidic Conditions

**Figure 3:**
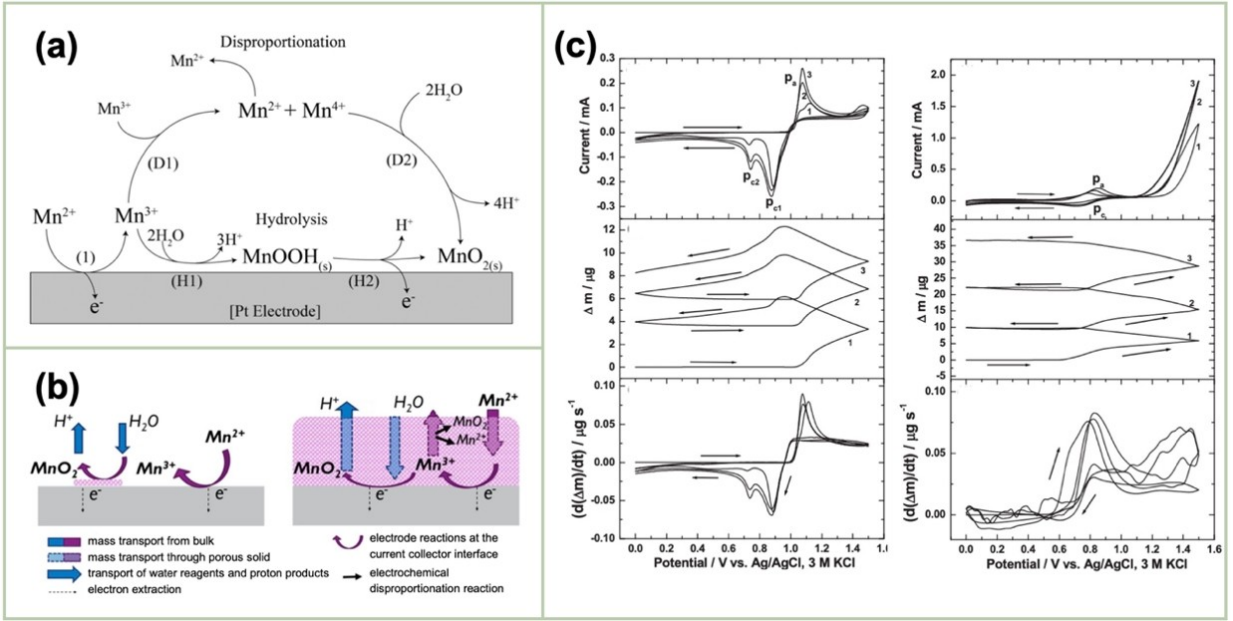
(a) Schematic of the oxidation of aqueous Mn^2+^ into solid MnO_2_. Reproduced with permission from Ref.^[42]^ Copyright 2020, Electrochemical Society. (b) Schematic of the MnO_2_‐growth mechanism. Reproduced with permission from Ref.^[43]^ Copyright 2017, Electrochemical Society. (c) EQCM responses recorded during the MnO_2_ deposition using (left) H_2_SO_4_ and (right) sodium sulfate (Na_2_SO_4_). Reproduced with permission from Ref.^[30]^ Copyright 2009, Electrochemical Society.

In an acidic electrolyte (Figure 3a), the electrodeposition commences with the diffusion of Mn^2+^ from the bulk solution to the electrode surface regardless of the solution pH (1′), followed by the oxidation of Mn^2+^_ads_ to Mn^3+^_ads_ (1).^[42]^ In the case of a highly acidic electrolyte, the generated Mn^3+^ undergoes hydrolysis to form MnOOH (H1). Thereafter, the generated H1 on the electrode surface is oxidized into MnO_2_ (H2.

(2)

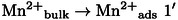



(3)

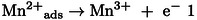



(4)

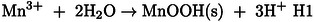



(5)

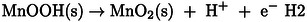




The above scheme is characteristic of the case where the acid concentration of the electrolyte is relatively low (<1 M) and in which the stability of the intermediate Mn^3+^ is low.^[40]^ Under strongly acidic conditions (>1 M), the Mn^3+^−Mn^3+^ collision induces disproportionation (DISP) into Mn^2+^ and Mn^4+^ (D1), which are subsequently transformed into MnO_2_ through the hydration of Mn^4+^ (D2). In both scenarios, the rate‐limiting step is identified as the diffusion of Mn^2+^ in the solution to the electrode surface (1′).
(6)

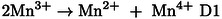



(7)

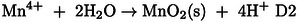




The MnO_2_ film predominantly grows at the substrate interface as the ionic species diffuse through the porous MnO_2_ film, as schematically shown in Figure 3b.^[43]^ Notably, MnO₂ is a poor electronic conductor; thus, it would rapidly passivate the electrode if it was formed as a dense film. However, the deposit is nanoporous, and the electrode reaction can proceed at the original electrode surface with slower ionic diffusion through the nanopores. Transport encompasses the movement of Mn^2+^ in the bulk toward the electrode surface, where the reaction occurs, as well as the diffusion of H^+^ out of the nanoporous MnO_2_ film. This mechanism accounts for the formation of thicker MnO_2_ films.

The electrooxidation reactions of Mn^2+^ into MnO_2_ by cyclic voltammetry on a gold electrode in acidic (0.1 M H_2_SO_4_) and neutral (0.1 M Na_2_SO_4_) media were studied using EQCM. The cyclic‐voltammetric behavior of Au varied between these electrolytes. The EQCM data of the mass variation during cycling revealed that the electrodeposition rate for MnO_2_ was higher in the neutral medium than in the acidic one (Figure 3c).^[30]^

#### 3.1.2 Neutral‐to‐Alkaline Conditions

As researchers have mostly explored the electrodeposition of EMD (γ‐MnO_2_) in strongly acidic solutions, only a few studies have explored MnO_2_ electrodeposition in neutral aqueous solutions. The mechanism of MnO_2_ deposition in a neutral electrolyte is markedly distinct from that described above, as evidenced by the distinctive crystalline structure of the resulting products. Scholars have generally confirmed that δ‐MnO_2_‐type layered MnO_2_ is formed in neutral solutions.[^31^, ^32^] Larabi‐Gruet *et al*.^[32]^ investigated the deposition of MnO_2_ in a neutral solution of 0.004 M MnSO_4_ and 0.4 M Na_2_SO_4_. The anodic oxidation of Mn^2+^ in a neutral, deaerated solution yielded a mixture of δ‐MnO_2_ and groutite (α‐MnOOH). This latter can be gradually oxidized into Mn^4+^, as follows:
(8)

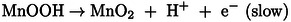




The presence of dissolved oxygen induced the formation of pure δ‐MnO_2_ exhibiting strong adhesion to a glass plate coated with tin oxide (SnO_2_). Batchelor‐McAuley *et al*. investigated MnO₂ electrodeposition at physiological pH levels.^[44]^ They proposed that the ECE process from intermediate species, i. e., two one‐electron‐transfer steps separated by a chemical step, and the DISP process compete at this point. At a low pH (<3), the DISP process becomes the dominant mechanism, whereas at a high pH (>7), proton diffusion from the intermediate MnOOH accounts for the rate‐determining step, facilitating the ECE process. Huynh *et al*. investigated the nucleation and steady‐state‐growth mechanism of MnO_x_ from Mn^2+^ in weakly alkaline electrolytes. The measured chronoamperometric transients fitted well with the progressive nucleation mechanism.^[45]^

Nakayama *et al*. employed the following reaction for the electrodeposition of δ‐MnO_2_ from aqueous MnSO_4_ solutions containing other cationic species (C^+^).^[31]^

(9)

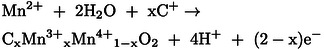




The reaction is distinguished by the incomplete oxidation of Mn^2+^, which can be regulated by an applied potential; moreover, the presence of Mn^3+^ furnishes a negative charge on the deposited MnO_2_, which is electrically neutralized by the intercalation of guest cations (C^+^) from the deposition bath. The veracity of the reaction (Equation (9)) was confirmed by the correlation of the interlayer distance of C_x_Mn^3+^_x_Mn^4+^_1–x_O_2_ with the size of C^+^. Furthermore, it can be inferred that the growth of MnO_2_ is sufficiently slow compared with the diffusion of C^+^. Nakayama *et al*. reported that the synthesized crystalline δ‐MnO_2_ exhibited zero activity for OER,[^46^, ^47^] agreeing well with the results obtained with MnO_2_ with the same crystalline structure but synthesized using various methods, as will be discussed in Section 5.

Wei *et al*. manipulated the crystalline structure of anodically electrodeposited MnO_2_ nanocrystals by introducing complexing agents into the electrodeposition baths.^[33]^ Further, they fabricated MnO_2_ nanocrystals exhibiting three distinct crystalline structures: ε‐MnO_2_ prepared from complex‐free solutions, defective rock‐salt MnO_2_ prepared from ethylenediaminetetraacetic acid (EDTA)‐containing solutions, and defective antifluorite MnO_2_ prepared from citrate‐containing solutions. The deposition of MnO_2_ was conducted under the following conditions: deposition current density, 100 mA cm^−2^; electrolyte pH value, 7.0; and electrolyte temperature, 70 °C. Dupont *et al*. combined EQCM, small‐angle X‐ray scattering, quasi in situ atomic force microscopy, and ex situ transmission electron microscopy to monitor MnO_2_ electrodeposition in the presence of citrate anions.^[42]^ MnO_2_ was deposited onto a Pt substrate at 1.3 V vs. SCE from solutions containing 0.01 M MnSO_4_, 0.1 M Na_2_SO_4_, and citrate at varying concentrations. The variation in the citrate concentration induced a change in the deposition mechanism, with the Mn^3+^ intermediate becoming more stable. The differing stability of Mn^3+^ impacted the morphology of the deposited material. Furthermore, the solution‐phase deposition of MnO_2_ from Mn^2+^, which corresponds to the charging process, has been frequently discussed in studies on aqueous Zn secondary batteries. These studies explored the deposition/dissolution of MnO_2_ itself as the cathode reactions and have been reviewed by Liu *et al*.;^[16]^ thus, they will not be discussed here in detail.

#### 3.1.3 Surfactant‐ and Template‐Mediated Depositions

**Figure 4:**
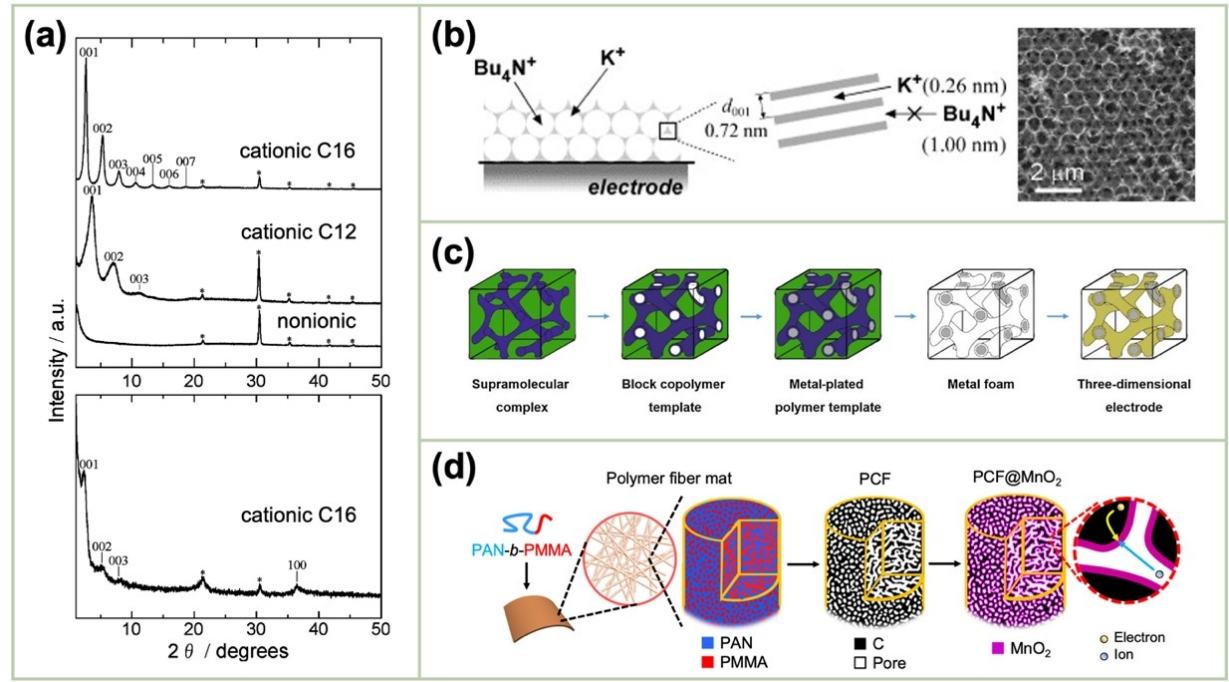
(a) (top) Out‐of‐plane and (bottom) in‐plane XRD patterns of the MnO_2_ deposited with the noted cationic surfactants. Reproduced with permission from Ref.^[50]^ Copyright 2010, American Chemical Society. (b) Schematic and scanning electron microscopy (SEM) image of the 3D‐ordered macroporous film of δ‐MnO_2_. Reproduced with permission from Ref.^[51]^ Copyright 2007, Elsevier. (c) Schematic of the synthetic procedure for the mesoporous MnO_2_ on the Ni foam (NF) fabricated using a block‐copolymer template. Reproduced with permission from Ref.^[52]^ Copyright 2016, Wiley‐VCH. (d) Schematic of the synthetic procedure for the mesoporous MnO_2_ on porous carbon fibers (PCFs). Reproduced with permission from Ref.^[53]^ Copyright 2019, Nature Research.

Lee *et al*. employed the anodic potentiostatic deposition method in a dilute surfactant solution of sodium dodecyl sulfate (SDS) to prepare meso‐structured lamellar MnO_2_ thin films.^[48]^ They observed the formation of the mesostructured thin films exclusively in the solution containing 0.1 M MnSO_4_ and 5 wt % SDS. The formation of the mesostructures could not be observed when cationic (cetyltrimethylammonium bromide (CTAB)) or nonionic copolymer surfactants (Brij56) were used. Osowiecki *et al*. demonstrated that MnO_2_ electrodeposited in the presence of SDS exhibited high activity and stability toward water oxidation in neutral media.^[49]^ Moreover, the deployment of surfactants during the anodic deposition of MnO_2_ by Nakayama *et al*. generated a distinct X‐ray diffraction (XRD) pattern.^[50]^ In this case, layered δ‐MnO_2_ was formed, with molecular layers of the surfactant sandwiched between the MnO_2_ layers. The layered structure was only formed in the presence of the cationic surfactants; i. e., cetyltrimethyl ammonium (C16) and dodecyltrimethyl ammonium (C12) (Figure 4a). The in‐plane XRD analysis confirmed the presence of crystallization within each MnO_2_ layer.

The continuous electrodeposition of MnO_2_ at a normal flat electrode will result in slow electronic conduction and ion diffusion in the thick MnO_2_ layer owing to its low conductivity and dense structure. The utilization of diverse templates for electrodeposition may prove an effective solution. A 3D‐ordered macroporous film of layered MnO_2_ was prepared by electrodeposition in the interstitial spaces of a template formed from polystyrene (PS) particles (diameter, 740 nm) on an indium–tin–oxide (ITO) electrode. Thereafter, the PS template was removed by dissolution in toluene, and the obtained ITO‐supported macroporous film exhibited excellent pseudocapacitive performance in a neutral electrolyte via the contributions of the macropore surface and interlayer space (<0.7 nm) of the multilayered structure (Figure 4b).^[51]^

Mesoporous MnO_2_ materials have been electrochemically deposited onto Pt‐coated Si substrates using triblock copolymer species (pluronic P123 and F127) as the structure‐directing agents.^[54]^ A binary system comprising 60 wt % surfactant P123 (or 50 wt % F127) and a 40 wt % (or 50 wt %) aqueous solution containing 0.5 M manganese(II) acetate (Mn(OAc)_2_) and 0.51 M potassium acetate was used as the electrolyte for the electrodeposition of the mesoporous MnO_2_. The electrodeposition was conducted at 1.0 V vs. SCE and room temperature. Following the deposition, the resulting electrode was immersed in 2‐propanol to remove the surfactant, followed by rinsing in high‐purity water and drying in ambient air. Tillmann *et al*. filled a porous nickel matrix with MnO_2_. The electrode was fabricated using a block‐copolymer template obtained from a 0.1 M MnSO_4_+0.1 M Na_2_SO_4_+0.1 M NaOAc solution (Figure 4c).^[52]^ The resulting electrode exhibited superior electrochemical performance as a high‐capacity lithium‐ion battery anode compared with the bulk counterpart. Liu *et al*. presented a design principle for carbon supports that utilize the self‐assembling and microphase separation of block copolymers.^[53]^ They synthesized porous carbon fibers (PCFs) with the uniform mesopores of 11.7 nm that were partially filled with MnO_2_ of <2 nm in thickness (Figure 4d). The uniform mesopores and ultrathin MnO_2_ facilitated rapid electron and ion transport compared with those observed in electrical double‐layer capacitive carbons.

### 3.2 Cathodic Deposition

Compared with anodic deposition, cathodic deposition has been barely studied.^[36]^ The cathodic deposition of MnO_2_ proceeds in two methods: the indirect and direct methods. The cathodic reduction of Mn does not occur in the former; rather, manganese(II)hydroxide (Mn(OH)_2_) is formed by the reaction between electrogenerated hydroxides (OH^−^) and Mn^2+^ in a solution.^[34]^ Conversely, permanganate anions (MnO_4_^−^) accept electrons directly from the electrode in the latter, resulting in the deposition of insoluble MnO_2_, as illustrated in the pH–*E* diagram in Figure 1.

#### 3.2.1 Indirect Method

The indirect method involves the formation of Mn(OH)_2_ at the cathode through a mechanism involving the electrogeneration of OH^−^ in a nitrate solution. This process is governed by a set of cathodic reactions, including the reduction of nitrate ions, dissolved oxygen, and water (Equation (10)–13):^[55]^

(10)

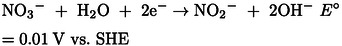



(11)

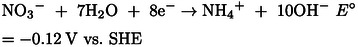



(12)

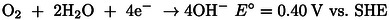



(13)

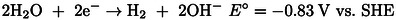




The above reactions increased the pH near the electrode surface. An increase in the OH^−^ concentration results in the formation of Mn(OH)_2_ (Equation (14)), which promotes deposition on the cathode.
(14)

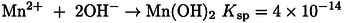




Moreover, Mn(OH)_2_ can be converted into MnO_2_ by annealing at 300 °C (Equation 15.
(15)

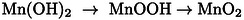




**Figure 5:**
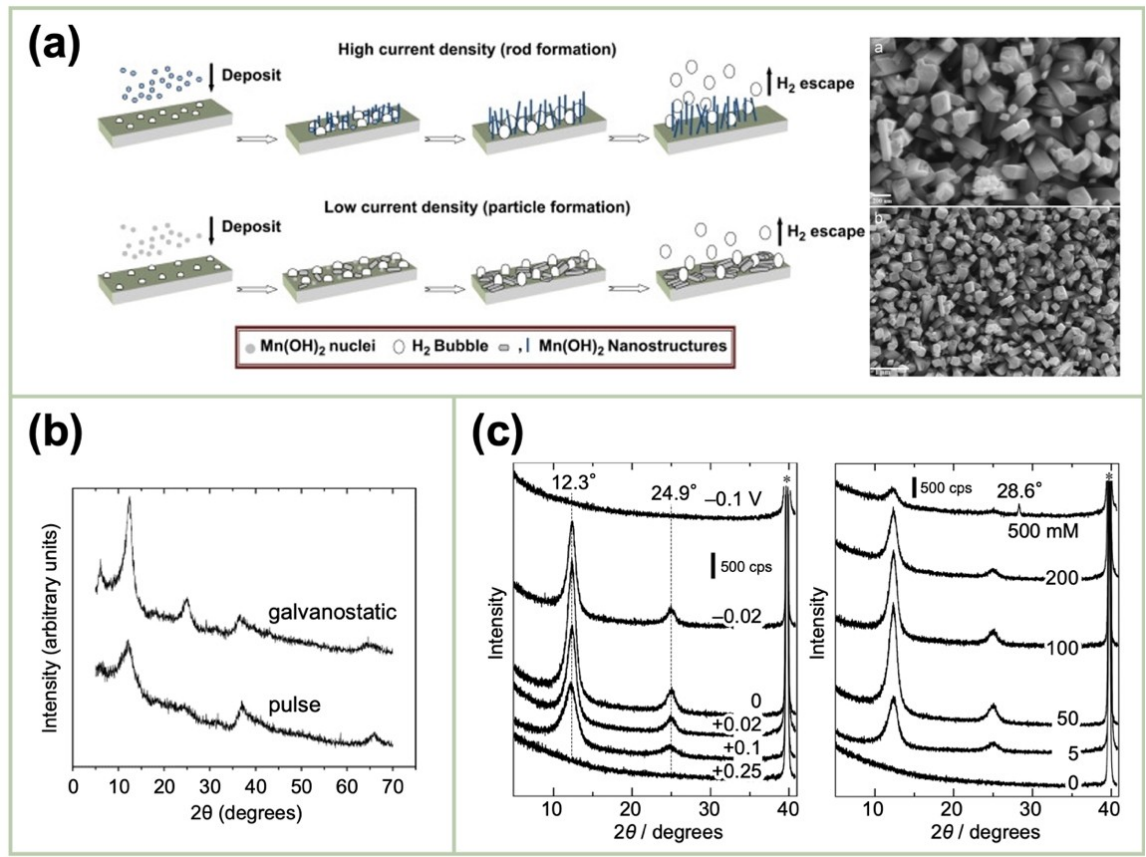
(a) Schematic of the formation of MnO_2_ particles at high and low current densities. Reproduced with permission from Ref.^[34]^ Copyright 2012, Elsevier. (b) XRD patterns of the MnO_2_ deposited from a KMnO_4_ solution via galvanostatic and pulse electrolysis. Reproduced with permission from Ref.^[35]^ Copyright 2008, Elsevier. (c) XRD patterns of the MnO_2_ deposited from a KMnO_4_ solution at varying applied potentials and concentrations. Reproduced with permission from Ref.^[36]^ Copyright 2012, Electrochemical Society.

Yousefi *et al*. employed a cathodic‐synthesis approach to produce Mn(OH)_2_ from a manganese(II) nitrate (Mn(NO_3_)_2_) solution at 10 °C, followed by annealing to obtain α‐MnO_2_.^[34]^ The resulting α‐MnO_2_ deposited at high and low current densities comprised vertically aligned nanorods and particles, respectively owing to the momentum of the generated hydrogen bubbles (Figure 5a).

#### 3.2.2 Direct Method

The pH–*E* diagram (Figure 1) shows that MnO_4_^−^ can be reduced into insoluble MnO_2_ (Equation 16.
(16)

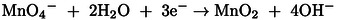




The principal advantage of the cathodic method is its avoidance of anodic corrosion as well as the dissolution of the electrode materials, which may occur in the anodic method. Jacob *et al*. revealed that nanostructured MnO_2_ films can be produced by galvanostatic, pulse, and reverse‐pulse electrodeposition from solutions containing 0.01–0.1 M KMnO_4_.^[35]^ The deposition yields were investigated by the in situ monitoring of the deposit mass using EQCM. The kinetic pathway of reducing Mn^7+^ to Mn^4+^ depends on the electrode potential, pH, MnO_4_^−^ concentration, and the presence of other species within the solution. The mass data indicated an increase in the deposition rate with the increasing KMnO_4_ concentration, indicating that the reaction was limited by the diffusion of MnO_4_^−^. Furthermore, the galvanostatic‐deposition‐obtained XRD pattern displayed a δ‐MnO_2_ phase (Figure 5b), agreeing with the findings of Nakayama *et al*. regarding the cathodic‐deposition‐prepared products at a constant potential from a MnO_4_^−^ solution. The crystallinity degree was influenced by the applied potential and concentration of the intercalated cations (Figure 5c).^[36]^

Gibson *et al*. elucidated the various steps of the electrodeposition mechanism of MnO_2_ from MnO_4_^−^.^[56]^ They proposed two primary mechanisms that depend on the applied potential and hydrodynamics. The initial step involves a combined reduction and DISP reactions, which include the reduction of Mn^VII^O_4_^−^ to Mn^VI^O_4_^2−^. This product subsequently undergoes hydrolysis and DISP, forming the Mn(V) species (H_2_Mn^V^O_4_^−^) that is further reduced to form Mn^IV^O_2_. This reaction persists across the entire potential range. However, the dynamic or static state of the system influences this phenomenon. In a dynamic system, an influx of MnO_4_^−^ facilitates continuous reaction, whereas in a static system, the lack of available MnO_4_^−^ hinders the progression of the reaction. The second mechanism entails the dissolution of pre‐existing MnO_2_ through an intermediate of MnOOH, yielding aqueous Mn^2+^. Subsequently, Mn^2+^ reacts with MnO_4_^−^ in the solution to yield MnO_2_. The aforementioned mechanisms have been corroborated by scanning electron microscopy (SEM) and X‐ray photoelectron spectroscopy (XPS).

### 3.3 Pulse Electrodeposition

The pulse electrodeposition technique has been used to synthesize single‐component MnO_2_ as well as MnO_2_‐based nanocomposites. The morphology of the deposited MnO_2_ can be altered by varying the pulse parameters, thereby enabling the manipulation of their electrochemical properties. Pulse polarization affects MnO_2_ and the scaffold structure.[^57^, ^58^]

**Figure 6:**
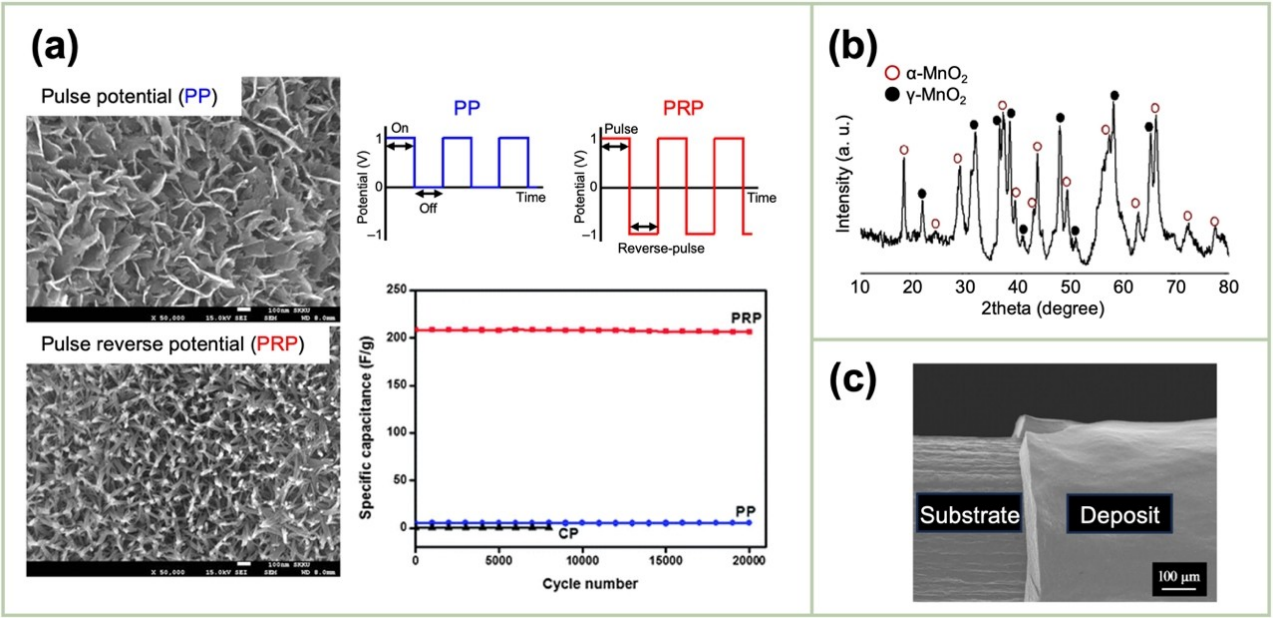
(a) SEM images of MnO_2_ prepared by pulse electrodeposition as well as its performance as a supercapacitor electrode compared with that prepared without pulsing. Reproduced with permission from Ref.^[59]^ Copyright 2013, Royal Society of Chemistry. (b) XRD pattern of MnO_2_ deposited by indirect cathodic method. Reproduced with permission from Ref.^[60]^ Copyright 2016, Elsevier. (c) SEM images of the deposits obtained by electrophoretic deposition (EPD). Reproduced with permission from Ref.^[63]^ Copyright 2008, Elsevier.

Lee *et al*. synthesized MnO_x_ nanostructures via the pulse‐reverse electrodeposition technique.^[59]^ The morphology and composition of the obtained MnO_x_ depended on the pattern of applying the potential. When a constant potential was applied, a bulk film comprising aggregated MnO_2_ particles was produced. However, the manganese(III)oxide (Mn_2_O_3_) nanostructures were formed using the pulse‐potential and pulse‐reverse‐potential (PRP) methods, with potential values of 1/0 and 1/−1 V, respectively. Moreover, the PRP method yielded a fine nanorod morphology, with high electrical conductivity, which ensured excellent capacitive performance (Figure 6a). Aghazadeh *et al*. performed the indirect cathodic electrodeposition of MnO_2_ from a 5 mM Mn(NO_3_)_2_ solution in a pulse current (−2 mA cm^−2^) mode with on‐time and off‐time (i. e., *t*_on_=1 s and *t*_off_=1 s, respectively). As already described (Section 3.2.1), the electrogeneration of OH^−^ at the electrode surface promoted Mn(OH)_2_ formation by associating with Mn^2+^. Following annealing at 400 °C, Mn(OH)_2_ was converted into MnO_2_ comprising α and γ phases (Figure 6b).^[60]^ Liu *et al*. employed pulse deposition for the synthesis of a polyaniline (PANI)/MnO_2_ composite film from an aniline + MnSO_4_ + H_2_SO_4_ solution (where the electropolymerization of aniline into PANI proceeded during the anodic deposition of MnO_2_).^[61]^ MnO_2_/reduced graphene oxide (GO), rGO, was synthesized by the in situ anodic pulse‐electrodeposition method, where current densities of 2 and 0.5 mA cm^−2^ (*t*_on_=10 ms) were applied repeatedly with a 30 s pause in a 0.1 M MnSO_4_+0.1 M Na_2_SO_4_ solution dispersed with 10 mg L^−1^ of GO nanosheets (NS). The electrodeposition of MnO_2_ facilitated the simultaneous incorporation of the GO colloids dispersed in the liquid. According to other researchers, the oxidative decarboxylation of the GO sheets facilitated the one‐step synthesis of the MnO_2_/RGO nanocomposite.^[62]^ Zhou *et al* used the pulse‐deposition technique to construct a TiO_2_@MnO_2_ nanotube (NT) array (NTA) structure. The titanium dioxide (TiO_2_) NTAs were subsequently immersed in a 0.25 M MnSO_4_+0.25 M Na_2_SO_4_ solution and subjected to an anodic pulse (100 mA cm^−2^, 0.5 s) with a sufficient relaxation time (0 mA, 10 s). During the anodic pulse, Mn^2+^ oxidation induced MnO_2_ formation, whereas the Mn^2+^ concentration in the NTs was restored during the subsequent pause. The homogeneous distribution of the MnO_2_ species in the NT layer was confirmed by elemental mapping using energy‐dispersive spectroscopy.^[57]^ Sumsudin *et al*. synthesized a TiO_2_ NT/Mn_2_O_3_ nanocomposite via cathodic‐pulse electrodeposition.^[58]^ They reported that Mn^2+^ and Mn^3+^ were reduced at −0.9 V vs. Ag/AgCl, with the reduced ions subsequently undergoing oxidation to become Mn^3+^, thereby promoting the accumulation of the Mn_2_O_3_ NPs within the NTs.

### 3.4 Electrophoretic Deposition

The electrophoretic deposition (EPD) technique leverages the electrophoretic phenomenon of charged colloidal particles to generate agglomerated layers of inorganic, polymer, and composite particles on an electrode substrate,[^14^, ^64^] following the application of an appropriate electric field between two electrodes to a solution of dispersed charged particles. The charged particles in the solution are transferred to the electrode exhibiting the opposite charge. Although electrodeposition represents a Faradaic process, EPD is a non‐Faradaic one. Accordingly, in the former, the loading mass of deposits can be estimated from the delivered electrical charge by considering Faradaic efficiency, although this does not apply to the latter. Accordingly, in EPD, the mass loading must be regulated by meticulous control, including the configuration of the two electrodes. EPD is straightforward and cost‐effective; it can be used to produce thin films of nanostructured particles over large areas without requiring binders. This makes it a highly scalable process with industrial suitability, similar to Faradaic electrodeposition. As previously outlined, EPD can effectively facilitate the formation of thin films comprising MnO_2_ particles at the anode and cathode. Notably, the deposition of the particles can be precisely regulated, with the thickness of the films easily controlled by the suspension concentration, applied potential, and deposition time.^[65]^ Nanostructured MnO_2_ particles, which have been synthesized, are dispersed in the EPD bath with and without additives that adsorb onto the MnO_2_ particles to stably charge and disperse them. As MnO_2_ particles do not typically undergo chemical reactions, their initial structure should be maintained. The EPD studies of MnO_2_ primarily focused on enhancing the performances of supercapacitor electrodes,[^63^, ^66^, ^67^] with other applications, including corrosion protection.^[68]^

Li *et al*. performed an EPD experiment using an aqueous suspension of MnO_2_ nanofibers at a concentration of less than 10 g L^−1[63]^ in the presence of alginate, a weak anionic polyelectrolyte (concentration, 0.1–0.5 g L^−1^). The MnO_2_ nanofibers were synthesized by the chemical reduction of MnO_4_^−^ using ethanol. The adsorption of alginate on the MnO_2_ nanofibers imparted it with an electric charge, which was a prerequisite for EPD. Notably, the application of a constant voltage of 5–50 V for 1–10 min facilitated the formation of anodic deposits on a variety of complex substrates, including a stainless mesh (Figure 6c). The film thickness can be modified by varying the applied voltage and EPD time. Further, a phosphate ester can be employed as an effective charging dispersant for the EPD of MnO_2_ films onto stainless steel (SS) substrates.^[66]^ Yousif *et al*. coated MnO_2_ NPs on Ni‐alloy cathodes using EPD to protect turbine blades from protection.^[68]^ The application of the EPD technology to the fabrication of MnO_2_ nanocomposites will be discussed in more detail in Section 4.

## 4 Electrodeposited Manganese Dioxide‐Based Composites

The performance of MnO_2_ is generally limited by its low conductivity (10^−5^ to 10^−6^ S cm^−1^) and relatively small accessible surface area to electrolyte and ionic species. These issues have been addressed by compositing pristine MnO_2_ with foreign metallic elements (dopants), carbon nanostructures, conducting polymers, or a combination thereof. The most facile method involves depositing the pristine MnO_2_ onto 3D porous scaffolds, such as carbonaceous materials or NFs, as alternatives to 2D metal plates with flat surfaces. This method facilitates the formation of pathways for electron and ion transfer. Carbon‐based freestanding scaffolds, such as graphene (GP) and carbon nanotubes (CNTs), have been employed in this context. They can be fabricated by filtering their dispersions. Most of these preparation strategies were developed for supercapacitor electrodes, although their application properties are only briefly captured in this review. Rather, only representative performances are listed in Table S1 of Supporting Information, as we primarily focused on the synthetic methods. The utilization of carbon‐based materials ensures improved conductivity and increased electrochemically active surface area, as well as enhances the flexibility of the supercapacitor devices.

### 4.1 Manganese Dioxide with Other Metals

Numerous studies have been conducted to improve MnO_2_ by incorporating it with foreign metals. One strategy for enhancing the electronic structure, introducing defects, and facilitating charge‐carrier generation is the incorporation of foreign metallic elements into the MnO_2_ structure. The binding energy between the catalyst and reactant can be modulated in the electrocatalysis context. Nakayama *et al*. proposed a methodology for introducing Co ions into the δ‐MnO_2_ framework through single‐step anodic deposition from a MnSO_4_+CoSO_4_ solution at 70 °C. During anodization, Co^2+^ was catalytically oxidized by the electrogenerated Mn^3+^ to form Co‐doped MnO_2_ deposits. Studies have revealed that the incorporation of Co enhances the electrochemical kinetics of MnO_2_, thereby ensuring excellent performance in various applications, including I^−^ detection,^[69]^ as cathode material for ZIBs,^[70]^ and as an electrode material for supercapacitors.^[71]^ Xiao *et al*. electrodeposited PtAu‐alloy and MnO_2_ nanocomposites onto a freestanding GP paper via potential cycling over a wide potential region between 1.4 and −1.5 V vs. Ag/AgCl in a MnSO_4_ solution containing K_2_PtCl_6_ and KAuCl_4_. Further, MnO_2_ was deposited at anodic potentials, whereas Pt and Au were deposited at cathodic potentials. The resulting PtAu–MnO_2_ electrode facilitated the amperometric detection of glucose.^[72]^

### 4.2 Manganese Dioxide Composites with Nanocarbons

The poor electrical conductivity (10^−5^ to 10^−6^ S cm^−1^) and low porosity of MnO_2_ have been improved by incorporating them with carbonaceous materials exhibiting good electrical conductivity. Although not via electrodeposition, MnO_2_ can be fabricated by allowing MnO_4_^−^ to contact with CNTs, as illustrated by the following equation. In this case, the electrons are transferred from the carbon surface to MnO_4_^−^.^[73]^

(17)

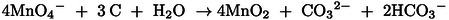




**Figure 7:**
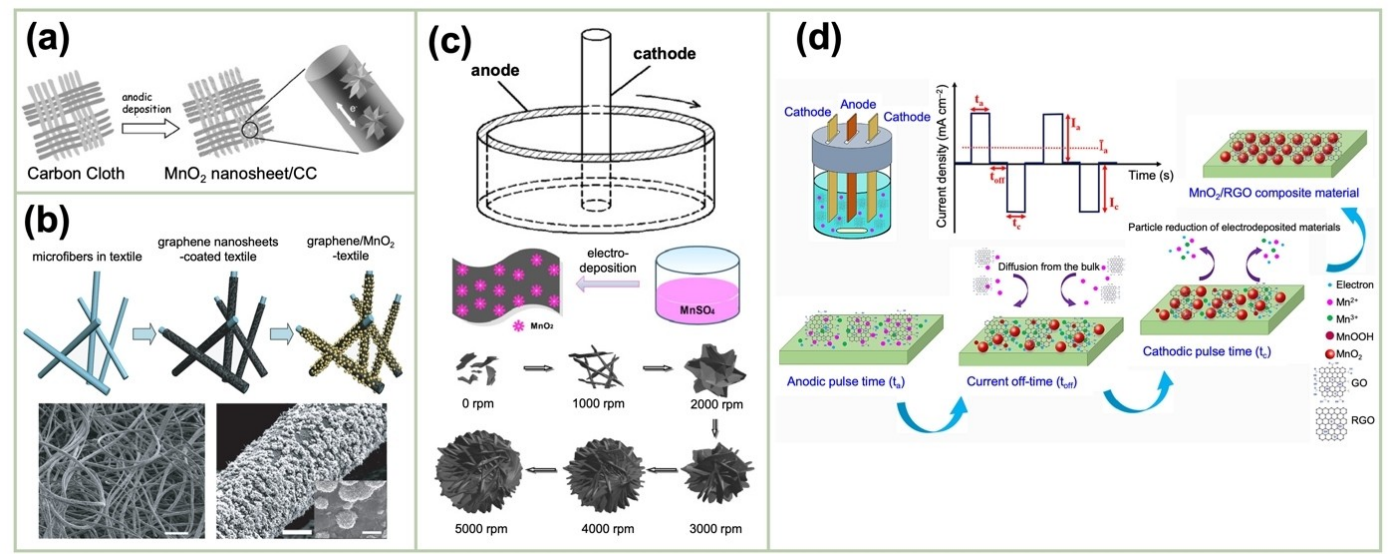
(a) Schematic of MnO_2_ electrodeposition on a CC electrode. Reproduced with permission from Ref.^[74]^ Copyright 2011, Elsevier. (b) Schematic of MnO_2_‐nanomaterial electrodeposition onto GPNS‐coated porous fibers and the SEM images of the product material. Reproduced with permission from Ref.^[78]^ Copyright 2011, American Chemical Society. (c) Schematic of MnO_2_ electrodeposition in a supergravity field and the relationship between the rotation rate and morphology of the MnO_2_ particles. Reproduced with permission from Ref.^[82]^ Copyright 2011, Elsevier. (d) Synthesis scheme of MnO_2_/RGO nanocomposites by pulse electrodeposition accompanied by the dissolution of the Mn species as well as reduction of GO into RGO at the cathode. Reproduced with permission from Ref.^[85]^ Copyright 2024, Elsevier.

This reaction is terminated when the carbon surface is fully covered with the generated MnO_2_; this reaction is self‐terminating. The resulting MnO_2_/CNT nanocomposite exhibited a remarkably high specific capacitance of over 5 F cm^−2^. This method can be used to optimize the application potential of MnO₂ attributable to its integration with CNTs. Nevertheless, to optimize the electrochemical performance per unit geometric area, the mass loading of the MnO_2_ deposits must be increased. Chen *et al*. reported a facile and cost‐effective method for synthesizing a hierarchically porous structure comprising δ‐MnO_2_ on a piece of carbon cloth (CC), where MnO_2_ was anodically deposited onto CC from a solution of 0.1 M H_2_SO_4_ and 0.1 M MnSO_4_ at a constant current density of 0.5 mA cm^−2^ (Figure 7a).^[74]^

#### 4.2.1 Graphene

The simplest compositing between MnO_2_ and GP is achieved by the anodic polarization of a freestanding GP paper in a Mn(OAc)_2_+Na_2_SO_4_ solution.^[75]^ The GP paper was obtained by the vacuum filtration of a GP‐NS dispersion, followed by drying. He *et al*. fabricated a 3D GP scaffold for the electrodeposition of a high mass loading of MnO_2_ (9.8 mg cm^−2^). The electrodeposition was conducted by applying current pulses to a Mn(NO_3_)_2_+NaNO_3_ solution.^[76]^ In this process, the high conductivity and porous structure of the 3D GP networks served as a dual conduit for electron transfer as well as facilitating the accessibility of the electrolyte ion to the electrode surface. The favorable contact between the GP and electrodeposition‐prepared MnO_2_ nanomaterials ensured low contact resistance and strong adhesion between them. Similarly, Chen *et al*. employed 3D‐GP‐coated fibers as a scaffold for the galvanostatic deposition of MnO_2_ at 0.4 mA cm^−2^ (equivalent to 0.7 V vs. Ag/AgCl) from a MnSO_4_+Na_2_SO_4_ solution.^[77]^ As illustrated in Figure 7b, Yu *et al*. coated GP NSs onto highly porous textile fibers on which MnO_2_ nanomaterials were electrodeposited via a facile and dry process.^[78]^ Further, a GO‐decorated glassy carbon (GC) electrode (GCE) was subjected to potential cycling between 0 and −1.5 V vs. Ag/AgCl in a Mn(OAc)_2_+Na_2_SO_4_ solution, and this resulted in the electrodeposition of MnO_2_ with a nanowall morphology as well as the reduction of GO into GP.^[79]^ The SEM images revealed that the MnO_2_ nanowalls were positioned on the GP surface. Veeramani *et al*. reported the facile electrochemical synthesis of an Au−MnO_2_ nanocomposite that was highly dispersed on an electrophoretically deposited GP surface.^[80]^ The addition of Au yielded nanoscale electrical contacts between the MnO_2_ nanowires and the GP surface. Xiong *et al*. proposed a procedure incorporating EPD, electrodeposition, and chemical vapor deposition (CVD) to fabricate a 3D MnO_2_–GP–CNT hybrid.^[81]^ The initial step involved the electrodeposition of MnO_2_ nanowalls onto a Ti substrate using a Mn(OAc)_2_+Na_2_SO_4_ solution. The resulting MnO_2_/Ti electrode was immersed in a GO‐NS dispersion and subsequently subjected to EPD for the anodic deposition of GO on it. The CNT growth on the MnO_2_/GO composite generated a high‐speed channel for carriers and imparted MnO_2_ with excellent pseudocapacitance. Liu *et al*. proposed a unique method for conducting pulse electrodeposition in a supergravity field to synthesize MnO_2_–GP composites.^[82]^ The deployment of supergravity facilitates enhanced mass transfer and mitigates concentration polarization during electrodeposition. Additionally, a MnSO_4_ solution containing a dispersed GO solution was subjected to anodic pulse electrolysis at a current density of 0.8 mA cm^−2^, during which the supergravity was regulated by modifying the rotation speed, as reflected in the morphology of the deposits (Figure 7c).

Additionally, an NF was employed as a scaffold for fabricating MnO_2_/RGO in the development of a binder‐free supercapacitor.^[83]^ The initial step involved the anodic deposition of MnO_2_ at a constant potential of 0.6 V vs. Ag/AgCl using a Mn(OAc)_2_+Na_2_SO_4_ solution. Subsequently, the resulting MnO_2_/NF was cathodically polarized in a GO‐dispersed solution, yielding MnO_2_/rGO on the NF scaffold. Notably, the GO NSs were reduced into rGO in conjunction with their electrophoretic deposition. Ren *et al*. recently coated 3D GP on the surface of an NF using the CVD technique.^[84]^ Subsequently, the 3D GP/NF electrode was subjected to polarization at 0.75 V vs. SCE in a solution comprising Mn(OAc)_2_ and Na_2_SO_4_ to yield a Na_0.11_MnO_2_/3DGP NF. Mahdi *et al*. reported the fabrication of an MnO_2_/rGO composite on an SS plate by applying anodic and cathodic pulse currents in a solution containing dispersed GO sheets.^[62]^ Anodic (2 mA cm^−2^) and cathodic (0.5 mA cm^−2^) pulse currents were applied to the SS plate in a MnSO_4_+Na_2_SO_4_ solution containing dispersed GO sheets. In the anodic pulse, MnO_2_ was deposited on SS in conjunction with GO incorporation. In the cathodic pulse, the SS‐supported GO was reduced to rGO. Additionally, a new MnO_2_/rGO composite was fabricated by varying the cathodic current density to a more negative value (−2 mA cm^−2^). The cathodic pulse was accompanied by the partial dissolution of MnO_2_, further improving the charge‐storage performance (Figure 7d).^[85]^ Purwaningsih *et al*. recently developed a two‐step electrochemical deposition process for combining γ‐MnO_2_ particles with rGO NSs.^[86]^ The resulting composite electrode was deployed as an ORR catalyst. Here, GO was electrophoretically deposited on an NF anode, after which γ‐MnO_2_ was cathodically deposited on the NF‐supported GO accompanied by the GO reduction into rGO. The fabricated electrodes exhibited remarkable ORR activity.

#### 4.2.2 Carbon Nanotubes

Chou *et al*. coated a CNT paper with MnO_2_ nanowires via anodic deposition using potential cycling between +0.60 and +0.3 V vs. SCE from a Mn(OAc)_2_ +0.1 M Na_2_SO_4_ solution.^[87]^ They obtained a flexible supercapacitor electrode exhibiting a large specific capacitance as well as good cyclability. The MnO_2_ nanowire was electrodeposited on a freestanding CNT paper at a constant potential of +0.7 V vs. Ag/AgCl from a Mn(OAc)_2_ solution.^[88]^ The aqueous ZIB assembled using the obtained MnO_2_/CNT paper cathode exhibited a high discharge capacity (292.7 mAh g^−1^@0.2 mA cm^−2^) that approached the theoretical value (308 mAh g^−1^), along with an enhanced rate capability (137 mAh g^−1^@3 mA cm^−2^). Furthermore, the material exhibited excellent long‐term cycling stability, retaining 85.72 % of its initial capacity after 1,000 cycles, and this was attributed to the enhanced electrochemical kinetics. In another study, MnO_2_/CNTs were constructed on an SS substrate by EPD in the presence of sodium alginate (SA) in a bath containing dispersed CNTs and MnO_2_ nanofibers.^[89]^ In the fabrication, SA, a polyelectrolyte, functioned as a dispersant, charging additive, and binder for the EPD of the MnO_2_ nanofibers and CNTs. Boccaccini *et al*. reviewed the EPD techniques for fabricating CNTs.^[90]^ Dopamine imparts MnO_2_ and CNTs with positive charges, thereby stabilizing them for cathodic deposition on the SS surface.^[91]^ Jin *et al*. electrodeposited MnO_2_ onto a flexible CNT paper from a MnSO_4_ + H_2_SO_4_ solution, after which GP was adsorbed onto the surface of the MnO_2_/CNT paper by dipping it into a GP‐dispersed solution.^[92]^ MnO_2_/carbon black (CB) nanocomposites were deposited from an aqueous SA solution containing MnO₂ NPs and CB dispersion onto a Ni plate by the EPD process.^[93]^ Both components were rendered negatively charged by SA, after which they accumulated on the anode. The SA deposited together was subsequently converted into carbon by heat treatment at 700 °C. Anionic humic acid also represents an effective dispersing agent in ethanol–water solvents, enabling the formation of MnO_2_/CNT composites during EPD, with a DC voltage of 100 V.^[94]^

### 4.3 Manganese Dioxide Composites with Conducting Polymers

**Figure 8:**
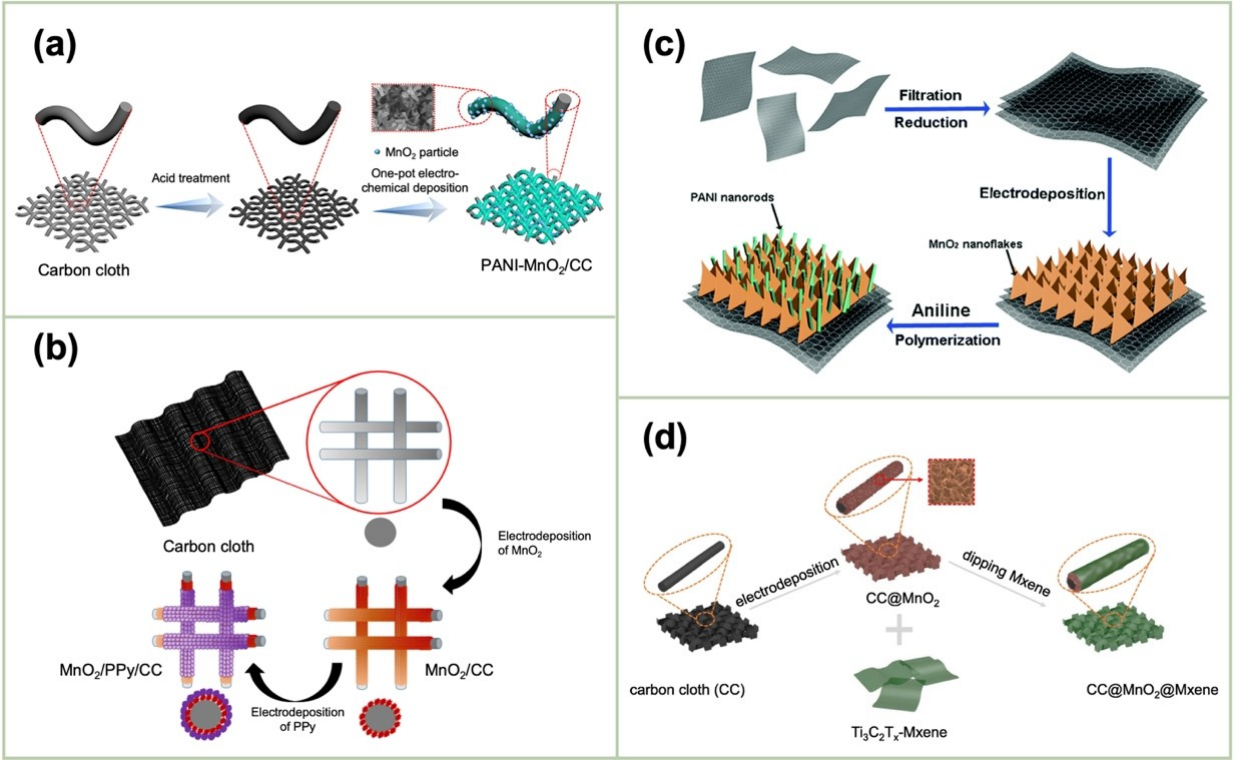
Schematic of the synthetic procedures. (a) One‐pot electrodeposition of MnO_2_/PANI nanocomposites on a CC substrate in the constant‐potential mode. Reproduced with permission from Ref.^[96]^ Copyright 2024, American Chemical Society. (b) Two‐step electrodeposition of MnO_2_/PPy nanocomposites on a CC substrate in the constant‐potential mode. Reproduced with permission from Ref.^[98]^ Copyright 2016, Elsevier. (c) Ternary composite comprising rGO, MnO_2_ nanoflakes, and PANI nanorods. Reproduced with permission from Ref.^[100]^ Copyright 2015, Royal Society of Chemistry. (d) MnO_2_/Ti_3_C_2_T_x_ MXene (MX) NSs on CC. Reproduced with permission from Ref.^[101]^ Copyright 2022, Elsevier.

Liu *et al*. fabricated a coaxial MnO_2_/poly(3,4‐ethylenedioxythiophene) (PEDOT) nanowire.^[95]^ The MnO_2_ and PEDOT nanowires were electrodeposited on Au‐sputtered nanoelectrodes exhibiting two distinct shapes (i. e., ring and flat‐top shapes, respectively) within the pores (diameter, 200 nm) of an anodized aluminum oxide template. PEDOT generally favors growth on the sharp edge of the ring‐shaped electrode, whereas MnO_2_ was more likely to be deposited on the flat‐top electrode probably owing to its smooth surface. Coaxial nanowires are formed by the simultaneous growth of the core MnO_2_ and shell PEDOT, as evidenced by the analysis of the current density obtained from electrochemical deposition. Further, MnO_2_ is a promising material for Zn‐storage cathodes when applied to ZIBs, exhibiting a high operating voltage and good rate performance in addition to its intrinsic properties, i. e., its high theoretical capacity (308 mA h g^−1^). As illustrated in Figure 8a, Xi *et al*. performed the one‐pot electrodeposition of MnO_2_/PANI on a CC substrate by applying a constant potential of +0.9 V vs. Ag/AgCl in an aqueous H_2_SO_4_ solution containing an aniline monomer and MnSO_4_.^[96]^ Further, Lee *et al*. fabricated 1D‐nanostructure‐based multiscale composites comprising MnO_2_ nanofibers (diameter, ~30 nm) on CC as energy‐storage devices.^[97]^ The surface of the MnO_2_, which had been electrodeposited from a Mn(OAc)_2_+Na_2_SO_4_ solution, was modified using partially carbonized polypyrrole (PPy) obtained from the electropolymerization and subsequent thermal annealing processes. The multiscale composite exhibited low resistivity and high rate capability. The high conductivity of the coating layer offered an effective pathway for charge transport. The absence of a binder resulted in a low interfacial resistance and a rapid electrochemical‐reaction rate. Fan *et al*. reported a high‐performance flexible electrode based on the electrodeposition of PPy/MnO_2_ on CC for supercapacitors.^[98]^ Initially, MnO_2_ was anodically deposited onto CC at a constant potential of 0.92 V vs. SCE from a Mn(OAc)_2_+Na_2_SO_4_ solution. Thereafter, the MnO_2_/CC electrode was subjected to polarization at 0.8 V in an aqueous NaClO_4_ solution containing a 0.2 vol% pyrrole monomer (Figure 8b). Liu *et al*. recently proposed a composite comprising carboxylated CNTs, α‐MnO_2_ nanorods, and PPy as a high‐capacitance electrode with commendable electrosorption capabilities for U(VI).^[99]^ The uranium‐containing wastewater, which was generated as a by‐product of the nuclear fuel cycle, has been identified as a significant environmental and public health hazard. The electrode was fabricated based on the potentiostatic electrodeposition of PPy onto the surface of carboxylated CNT/α‐MnO_2_ nanorod composites.

### 4.4 Ternary Composites

Ng *et al*. synthesized PPy/GO/MnO_x_ nanocomposites on NFs.^[102]^ The nanocomposites were electrodeposited via a one‐pot, one‐step approach by applying a constant potential of +0.8 V. The deposition bath comprised a 0.1 M pyrrole monomer, 1 mg mL^−1^ GO, 0.1 M toluene‐4‐sulfonic acid sodium salt, and 0.1 M MnSO_4_. Li *et al*. also employed a two‐step methodology to fabricate a freestanding rGO paper modified with MnO₂ nanoflake/PANI nanorods for application in flexible supercapacitor electrodes (Figure 8c).^[100]^ The MnO_2_ nanoflakes were synthesized on the rGO paper via potentiostatic electrodeposition (0.8 V vs. Ag/AgCl) from an Mn(OAc)_2_+Na_2_SO_4_ solution. Subsequently, PANI nanorods were assembled between MnO_2_ nanoflakes via in situ polymerization using camphorsulfonic acid as a dopant. Toumsi *et al*. directly prepared a ternary composite film comprising PANI, MnO_2_, and GP on a fluorine‐doped tin oxide (FTO) glass substrate (FTO/PANI–MnO_2_–GR).^[103]^ The film was obtained by the electropolymerization of aniline in conjunction with the electrodeposition of MnO_2_; both processes proceeded simultaneously under the same applied potential of 1.2 V vs. SCE. A MnSO_4_ + Na₂SO_4_ + aniline monomer solution dispersed with GP NSs was used as the deposition bath. MnO_2_ and PANI were simultaneously deposited at the applied potential accompanied by the incorporation of the GP NSs.

### 4.5 Other Composites

In a recent study, Qi *et al*. fabricated a flexible electrode comprising 3D interconnected ultrathin MnO_2_ NSs on CC coated with Ti_3_C_2_T_x_ MXene (CC@MnO_2_@MX) by electrodeposition and dipping methods.^[101]^ As illustrated in Figure 8d, the initial step involved the anodic deposition of MnO_2_ onto a CC substrate at a constant current of 1 mA cm^−2^. The resulting MnO_2_/CC was impregnated with an MX dispersion to facilitate the complete wrapping of MnO_2_/CC with MX. An aqueous ZIB battery assembled with the MX/MnO_2_/CC cathode demonstrated high charge‐storage performance (517.0 mAh g^−1^@0.1 A g^−1^), excellent cycling stability (80.6 mAh g^−1^ after 800 cycles @1 A g^−1^), and superior energy density (701.3 Wh kg^−1^ @133.8 W kg^−1^). The excellent electrochemical performance is primarily attributable to the highly conducting MX coating, which generated ion and electron pathways for the MnO_2_ NSs (thereby reducing the length of the ion‐ and electron‐transport paths) and enhancing the conductivity of the composite.

## 5 Electrocatalysis

In this chapter, the electrocatalysis of the electrochemically grown Mn oxides on substrate electrodes is described. Electrodeposition‐prepared materials are advantageous to chemically synthesized powder materials, as they are not combined with polymer binders, such as Nafion or polytetrafluoroethylene. Polymer binders increase the charge‐transfer resistance and obstruct the ion‐diffusion pathways. Furthermore, the clean environment and thin‐film form are more compatible with various operando measurements, as they do not contain any additives.

Similar to the development of supercapacitor electrodes, it is anticipated that composites comprising conducting and porous substances will be advantageous in electrocatalysis. The introduction of foreign metallic elements also modifies the electronic structure of pristine MnO_2_, thereby enhancing its conductivity and reactivity. This section commences with an examination of OER electrolysis using electrodeposited single‐component MnO_2_, after which the catalytic properties of MnO_2_‐based composites were investigated and reported. The subsequent sections address ORR catalysts as well as OER/ORR bifunctional catalysts for FCs and metal–air batteries, which are operated by O_2_‐related reactions.

### 5.1 Oxygen Evolution Reaction

The production of green hydrogen via water electrolysis using renewable‐energy sources, such as solar and wind power, is pivotal to achieving carbon neutrality. It entails converting renewable‐energy sources into hydrogen, a clean chemical fuel with a high energy density (140 MJ kg^−1^).^[104]^ The overall water splitting reaction represents a thermodynamically uphill reaction (Δ*G*°=237 kJ mol^−1^, equivalent to 1.23 V). HER does not account for the limiting step in water splitting at the cathode; rather, OER at the anode accounts for it. This is because the OER represents a complex reaction that is accompanied by the transfer of four protons and electrons each as well as the formation of an O−O double bond, as expressed by the following reaction steps.^[105]^

In an alkaline electrolyte:
(18)

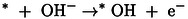



(19)

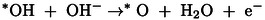



(20)

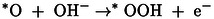



(21)

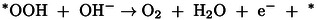




In an acidic electrolyte:
(22)

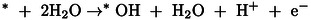



(23)

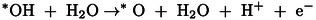



(24)

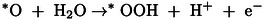



(25)

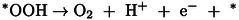




where * denotes active sites on the surface of the catalyst. All the intermediates are bound to the active sites on the catalyst surface. The activation barrier to the next state and the relative stability of these intermediates determine the rate‐determining step, as a result affecting the kinetics of OER. The characteristics of the intermediates–active site are contingent upon the reaction conditions and the catalyst species.[^106^, ^107^] The high OER efficiency of the biological system (photosynthesis II) has prompted extensive studies of the role of Mn oxides as an OER catalysts.^[5]^ The factors governing this study include the wide distribution of Mn oxides, their cost‐effectiveness, and possible excellent electrochemical properties.^[108]^ However, the majority of crystalline MnO_2_ polymorphs exhibit limited electrocatalytic OER activity, which is attributed to their poor conductivity, perfect lattice structure, and half‐filled t_2g_ orbitals.^[109]^ This section addresses the use of electrodeposited MnO₂ and its composites in catalytic OER reactions. It discusses the active site, Mn‐oxidation state, adsorption energy, and other pertinent factors. Notably, seawater electrolysis, which has recently attracted considerable interest, was performed over two decades ago by combining the electrodeposited MnO_2_ with other metals.

The synthetic procedures employed as well as the representative performance data obtained are presented in Table 3.

**Table 3:** Synthetic procedures as well as OER properties of reported MnO_2_ and MnO_2_‐based nanocomposites prepared by electrodeposition.^[a]^

Synthesis					Electrochemical test				Ref.
Deposit on substrate	Substrate	ED bath composition	ED procedure	Post‐treatment	Test electrolyte	Test condition	Overpotential@reacehed current	Tafel slope	
*γ*‐MnOOH nanorods	GC, Au, Pt	0.1 M Mn(OAc)_2_+0.1 M Na_2_SO_4_	Potential cycling (0–0.4 V vs. Ag/AgCl)		0.5 M KOH	20 mV s^−1^			[110]
MnO_x_	FTO	0.5 mM MnCl_2_+0.9 M KNO_3_+50 mM methylphosphonate	Constant potential (1.1 V vs. SHE) Potential cycling (1.7 to −0.8 V) Multipotential step (1.1, −0.4 V)		0.10 M phosphate‐buffered saline (PBS) 1.73 M KNO_3_ (pH 2.5)			123 mV dec^−1^ 68 mV dec^−1^ 67 mV dec^−1^	[111]
MnO_x_	ITO	0.5 mM Mn(OAc)_2_+0.1 M MgSO_4_+0.1 M NaOAc/HOAc	A: stepwise (1.4 to −0.25 V vs. SHE) B: stepwise (2.15 to −0.75 V) C: cycling (2.15 to −0.75 V)		0.1 M PBS (pH 7)	20 mV ‐s^−1^	565 mV@0.5 mA cm^−2^ 590 mV@1 mA cm^−2^ (with catalyst obtained by Protocol C)	0.01 s^−1^ per deposited Mn ion	[112]
MnO_2_	FTO	0.5 mM MnCl_2_+0.9 M KNO_3_	Multipotential steps (+1.1 V, −0.4 V vs. Ag/AgCl)		0.5 mM Mn^2+^+0.9 M KNO_3_	100 mV ^−1^			[113]
MnO_2_nanowires	NF	0.1 M MnCl_2_+Na_2_SO_4_	Potential cycling (0–0.6 V vs. Hg/HgO)		1 M KOH	10 mV s^−1^	260 mV	47 mV dec^−1^ (135 mV dec^−1^ for bare NF)	[114]
MnO_x_	FTO	10 mM Mn(OAc)_2_+1 M EAN (or NaNO_3_) pH 1.8	Constant current (0.2 mA cm^−2^)	Heating at 50 °C–120 °C	1 M NaOH	5 mV s^−1^		with EAN: 114 mV dec^−1^ (with NaNO_3_: 127 mV dec^−1^)	[115]
Mn_2_O_3_ MnO_x_‐573 K Mn_3_O_4_	FTO	0.25 M MnSO_4_+0.25 M Na_2_SO_4_	Constant current (0.25 mA cm^−2^)	Annealing at different temperatures	1 M KOH	2 mV s^−1^	α‐Mn_2_O_3_ 170 mV @0.1 mA cm^−2^ MnO_x_‐573 K 230 mV@0.1 mA cm^−2^ Mn_3_O_4_ 290 mV@10 mA cm^−2^		[117]
MnO_x_	FTO	0.5 mM MnCl_2_+0.9 M KNO_3_	Multipotential step (0.9 to −0.6 V vs Ag/AgCl) 405 nm illumination		0.1 M KOH+0.9 M KNO_3_	20 mV s^−1^		64.1 mV dec^−1^(72.7 mV dec^−1^ for MnO_x_ deposited in the dark)	[118]
MnO_2_	FTO	25 mM Mn(OAc)_2_+25 mM SDS	Potential cycling (2.15 to −0.75 V vs. SHE)		0.1 M PBS	20 mV s^−1^	440 mV@0.5 mA cm^−2^		[49]
δ‐MnO_2_ Mn_2_O_3_ Mn_3_O_4_	FTO	0.01 M Mn(OAc)_2_+EAN at 120 °C	Constant current (0.2 mA cm^−2^)		1 M NaOH	0.2 mV s^−1^		63 mV dec^−1^	[119]
P‐(PbO_2_–MnO_2_)	Lead	35 g L^−1^ Na_4_P_2_O_7_+16 g L^−1^ Pb(NO_3_)_2_+2 g L^−1^ Cu(NO_3_)_2_+1 g L^−1^ NaF+0.2 g L^−1^ peptone (pH ~11) at 70 °C 1) dispersing *γ*‐MnO_2_ particles 2) adding Mn(NO_3_)_2_	Constant current (10 mA cm^−2^)		1.63 M H_2_SO_4_	0.2 mV s^−1^	P‐PbO_2_ 615 mV P‐(PbO_2_–MnO_2_) 602 mV (P‐PbO_2_)–MnO_2_ 476 mV@50 mA cm^−2^	P‐PbO_2_ 257 mV dec^−1^ P‐(PbO_2_–MnO_2_) 260 mV dec^−1^ (P‐PbO_2_)–MnO_2_ 196 mV dec^−1^	[120]
Fe‐MnO_x_	FTO	1.5 mM KMnO_4_+1.0 mM Fe(NO_3_)_3_	Constant current (0.25 mA cm^−2^)		0.10 M PBS (pH 7)	20 mV s^−1^			[121]
metal‐ion (Fe, V, Co, and Ni)‐doped MnO_2_ ultrathin NSs *γ*‐MnO_2_	CFP	0.20 M MnSO_4_+ 0.10 M NH_4_Fe(SO_4_) +0.01 M NaVO_3_ +0.05 M Co(NO_3_)_2_ +0.01 M NiSO_4_ +1.1 M H_2_SO_4_ at 98 °C	Constant current (3 mA cm^−2^)		1 M KOH	20 mV s^−1^	390 mV	104 mV dec^−1^	[122]
Pristine MnO_2_		0.20 m MnSO_4_+1.1 m H_2_SO_4_ at 98 °C.	Constant current (3 mA cm^−2^)				467 mV	111.7 mV dec^−1^	
GO/MnO_2_–NiO	SS	0.02 M Mn(OAc) +0.05 M Ni(OAc) +0.5 M H_2_SO_4_ or 1.0 M Na_2_SO_4_	Potential cycling (0–1.4 V vs. Ag/AgCl)	Annealing at 300 °C–400 °C	0.1 M KOH	10 mV s^−1^	379 mV@10 mA cm^−2^	47.84 mV dec^−1^	[123]
MnO_x_–Ti_3_C_2_T_x_@TiO_2_/molybdenum disulfide (MoS_2_)	NF	0.1 M Mn(OAc)_2_	Constant voltage (1.4 V) conglutinated Ti_3_C_2_Tx@TiO_2_/MoS_2_	Annealing at 250 °C in the air	1 M KOH	10 mV s^−1^	270 mV@10 mA cm^−2^	118.62 mV dec^−1^	[124]
Co/MnO_2_	FTO	2 mM MnSO_4_+50 Bu_4_NCl	Constant potential (1.0 V vs. Ag/AgCl)	Immersion in 0.5 M Co_2_SO_4_	1 M KOH	1 mV s^‐1^		60 mV dec^−1^	[47]
MX–Ni_0.075_Mn_0.925_O_2_ (NMO)/CC	CC	1.69 g MnSO_4_ + Ni(NO_3_)_2_ + 1.42 g Na_2_SO_4_ + 0.28 mL H_2_SO_4_ in 50 mL water	Constant current (3 mA)	Dipping in an MX solution and polarizing at 5 V	1 M KOH	5 mV s^−1^	410 mV@50 mA cm^−2^	165 mV dec^−1^	[125]

[a] ED: electrodeposition, GC: glassy carbon, FTO: fluorine‐doped tin oxide, EAN: ethylammonium nitrate, SDS: sodium dodecyl sulfate, CFP: carbon fiber paper, SS: stainless steel.

#### 5.1.1 Single‐Component Manganese Dioxide

**Figure 9:**
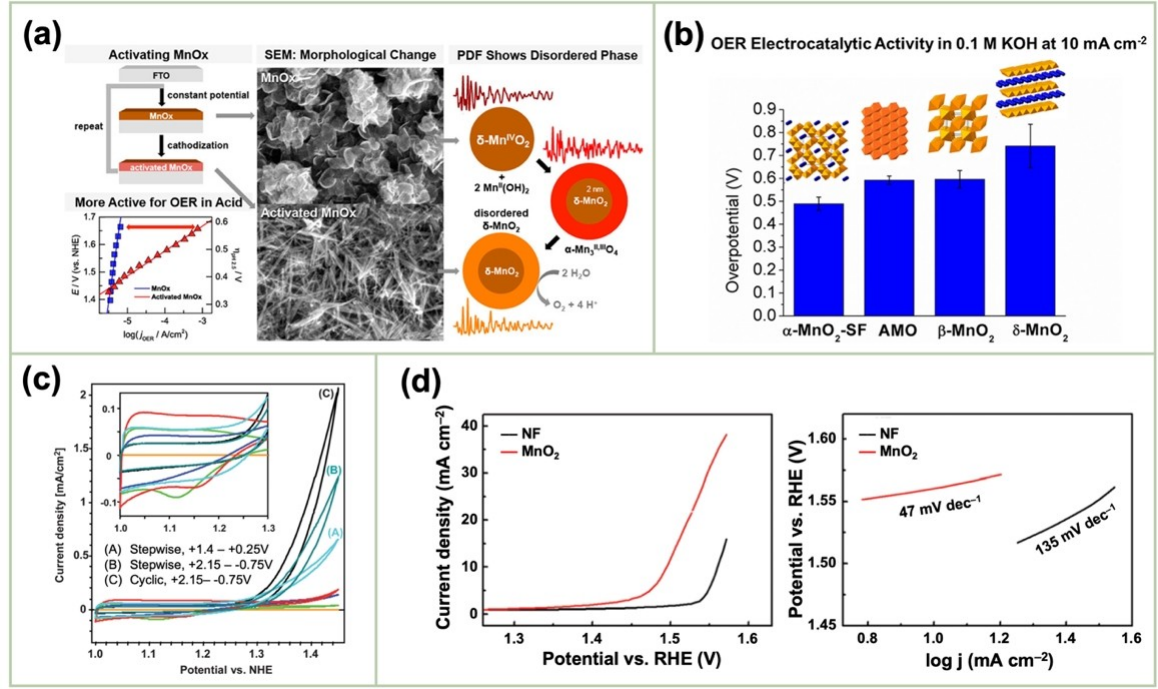
(a) Activation of inactive MnO_x_ deposited potentiostatically by cathodic polarization, including the Tafel plots and SEM images. Reproduced with permission from Ref.^[111]^ Copyright 2015, American Chemical Society. (b) OER overpotentials of various MnO_2_ crystalline polymorphs obtained chemically. Reproduced with permission from Ref.^[7]^ Copyright 2014, American Chemical Society. (c) OER characteristics of MnO_x_ after a change in the potential in various modes. Reproduced with permission from Ref.^[112]^ Copyright 2012, Royal Society of Chemistry. (d) OER characteristics of MnO_2_‐modified and unmodified 3D NFs. Reproduced with permission from Ref.^[114]^ Copyright 2020, Royal Society of Chemistry.

El‐Deab *et al*. deposited MnO_x_ NPs from a 0.1 M Mn(OAc)_2_ + 0.1 M Na_2_SO_4_ solution onto Au, Pt, and GC rotating‐disk electrodes (RDEs) via potential cycling between 0 and 0.4 V vs. Ag/AgCl.^[110]^ The resulting nano‐γ‐MnOOH exhibited enhanced catalytic activity toward OER owing to the improved electron transfer at various stages of the reaction pathway. Gorlin *et al*. reported the excellent OER activity of potentiostatically deposited MnO_x_ in an alkaline electrolyte.^[6]^ The observed OER performance indicated that the MnO_x_ nanostructure facilitated the formation of suitable Mn_x_O_y_ active sites at the applied potentials. Huynh *et al*. observed that the OER activity of a MnO_x_ film prepared at a constant anodic potential could be enhanced by subjecting it to anodic–cathodic pulse sequences (Figure 9a).^[111]^ They attributed this improvement to the formation of disordered δ‐MnO_2_, which is active toward OER. The MnO_x_ films prepared via multipotential electrodeposition (in which the Mn ions exist in a mixture of +3 and +4 oxidation states) exhibited a structure resembling δ‐MnO_2_, although with high disorder between layers of edge‐sharing MnO_6_ octahedra. These disordered films exhibit higher activity than the ordered δ‐MnO_2_ films electrodeposited at a constant potential.[^109^, ^111^, ^126^] The poor OER activity of crystalline δ‐MnO_2_ is also evident from comparative studies of various chemically synthesized MnO_2_ crystals (Figure 9b).^[7]^ The higher OER activity was ascribed to several factors, including mixed valences, abundant di–μ–oxo bridges in α‐MnO_2_, and low charge‐transfer resistances. Zaharieva *et al*. demonstrated that the selection of a potential protocol can transform an OER‐inactive MnO_x_ into a highly active catalyst.^[112]^ The electrodeposition at constant potential yielded an inactive MnO_x_, correlating with the observations in several studies.^[111]^ Conversely, potential cycling protocols resulted in an especially active catalyst. The X‐ray absorption spectroscopy (XAS) measurements revealed that the potential cycling protocol effectively inhibited the formation of stoichiometric Mn^IV^O_2_. The disorder in the atomic structure of the active MnO_x_ might facilitate μ_2_‐O(H) bridging as well as the terminal ligation of water, thereby enhancing its catalytic activity. Villalobos *et al*. observed a similar phenomenon. They galvanostatically deposited a Na‐containing MnO_x_ film from a 0.6 M Mn(NO_3_)_2_+6 M L‐(+)‐tartaric acid solution. The Tafel slope of the deposited film decreased with increasing the number of potential cycles in 0.1 M NaOH, which corresponds to the activation of the initial MnO_x_ film.^[127]^ Morgan Chan *et al*.’s in situ electrochemical and XAS studies demonstrated that the incorporation of Mn^3+^ into the lattice of MnO_2_ polymorphs significantly enhanced their OER activities.^[109]^ Additionally, the introduction of Mn^3+^ could be achieved by the potential cycling of the electrode coated with δ‐MnO_2_‐like MnO_2_ polymorphs.[^110^, ^112^] The XAS measurements conducted during the electroactivation of the deposited δ‐MnO_2_ indicated that the Mn^3+^ characteristic remained in the OER active catalysts and that the coordination number of the Mn−O bond was lowered, following the formation of Mn^3+^.[^109^, ^113^] The computational studies, which are supported by extended X‐ray absorption fine structure results, revealed two key findings: first, Mn^3+^ was stabilized at the tetrahedral sites; second, its presence strained the oxide lattice. On the other hand, Jin *et al*. used surface interrogation scanning electronmicroscopy to show that the OER sites on amorphous MnO_x_ that was deposited at a constant potential (+1.2 V vs. Ag/AgCl) from a 0.5 mM Mn(OAc)_2_+0.2 M sodium borate buffer solution (pH 9) always produced Mn^5+^ species, in addition to Mn^3+^ and Mn^4+^.^[128]^

Raut *et al*. fabricated MnO_2_ nanowires on 3D NF from a MnCl_2_+Na_2_SO_4_ solution by applying potential cycling between 0 and 0.6 V to develop materials for supercapacitor and electrocatalysis applications.^[114]^ Figure 9d shows that the MnO_2_‐nanowire electrode exhibited a relatively low Tafel slope of 47 mV dec^−1^ compared with NF, which exhibited a higher value (135 mV dec^−1^), indicating that the MnO_2_‐nanowire electrode displayed enhanced OER performance. Furthermore, the electrode exhibited the highest specific capacitance (641 F g^−1^) as well as excellent cycling properties, retaining 94.21 % of its initial capacity after 2000 cycles. The bifunctionality observed here indicated that OER catalysts and electrode materials for supercapacitors can be designed using the same strategy.

**Figure 10:**
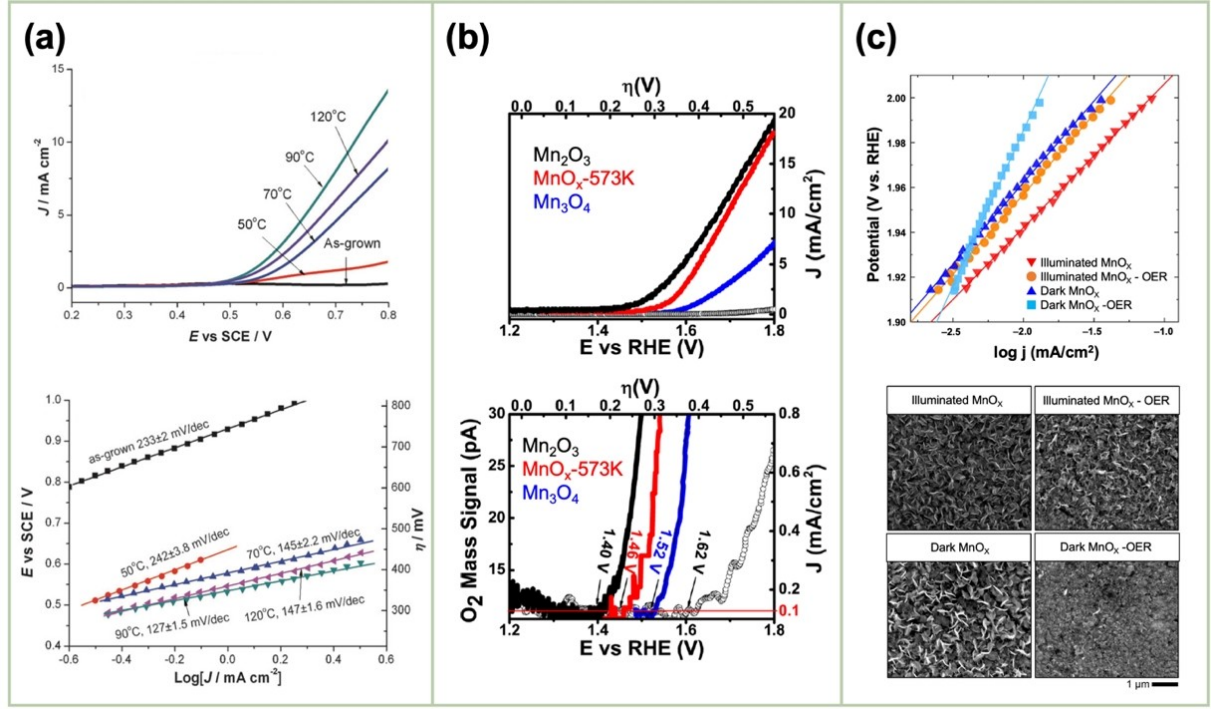
OER characteristics analyzed in alkaline solutions. (a) MnO_x_ films deposited at a constant current and heat‐treated at temperatures of up to 120 °C. Reproduced with permission from Ref.^[115]^ Copyright 2013, Wiley‐VCH. (b) Nearly amorphous MnO_x_ films deposited at a constant current and heat‐treated at temperatures of up to 600 °C. Reproduced with permission from Ref.^[117]^ Copyright 2014, American Chemical Society. (c) MnO_x_ films electrodeposited under light irradiation and in the dark, as well as those obtained after the respective OER tests together with the corresponding SEM images. Reproduced with permission from Ref.^[118]^ Copyright 2023, American Chemical Society.

Moreover, subjecting the electrodeposited MnO_x_ films to heat treatment significantly enhanced their OER activity. For example, Zhou *et al*. reported that the MnO_x_ films deposited from an aqueous bath comprising Mn(OAc)_2_+NaNO_3_ at pH 6 were simply activated for OER by heat treatment at temperatures of less than 120 °C (Figure 10a).^[115]^ The dehydration process was aimed at removing structural water and the hydroxyl species, thereby improving the conductivity and yielding a more active catalyst. This, in turn, contributes to the enhancement of water oxidation performances. Moreover, XAS revealed the formation of small amounts (3 %–10 %) of reduced Mn species (Mn^2+^, Mn^3+^) after the heat treatment.[^115^, ^116^] Ramilez *et al*. demonstrated how annealing significantly enhanced the OER activity of the nearly amorphous MnO_x_ film in a KOH solution.^[117]^ The initial MnO_x_ film was anodically deposited onto an FTO substrate from a MnSO_4_+Na_2_SO_4_ solution at a constant current density of 0.25 mA cm^−2^. The annealing of the material at 573, 773, and 873 K in the air and an inert atmosphere (nitrogen) resulted in the formation of crystalline structures as well as the conversion of the material into α‐Mn_2_O_3_ and Mn_3_O_4_, respectively. Figure 10b shows that α‐Mn_2_O_3_ (predominantly Mn^3+^) exhibited the highest OER activity, followed by MnO_x_‐573 K (Mn^3+^, Mn^4+^) and the least active catalyst, Mn_3_O_4_ (Mn^2+^, Mn^3+^). Together with a detailed structural analysis, including XPS and Raman spectroscopy, the authors established that the high catalytic activity for OER was attributable to the presence of Mn^3+^ in a distorted lattice, the high concentration of oxygen point defects, and a large variety of Mn−O bond lengths. Recently, Qin *et al*. demonstrated that the electrodeposition of Mn oxide under illumination facilitated the formation of nanostructured films with enhanced stability.^[118]^ These films exhibited a potential range of +0.9 to −0.6 V vs. Ag/AgCl, indicating their multipotential nature. Their OER activities were compared with those of films grown under otherwise identical conditions in the dark. The MnO_x_ film grown under illumination exhibited a Tafel slope of 60 mV dec^−1^, whereas that grown in the dark exhibited a slope of 72.7 mV dec^−1^. Following galvanostatic electrolysis at 5 mA cm^−2^ for 40 min in a 0.1 M KOH+0.9 M KNO_3_ solution, the Tafel slope of the illuminated MnO_x_ became 71.4 mV dec^−1^, whereas that of the dark film was 138 mV dec^−1^, which was not stable during the OER process, as also evidenced by the morphological changes in the SEM images (Figure 10c).

#### 5.1.2 Unconventional Manganese Dioxide and Composites

**Figure 11:**
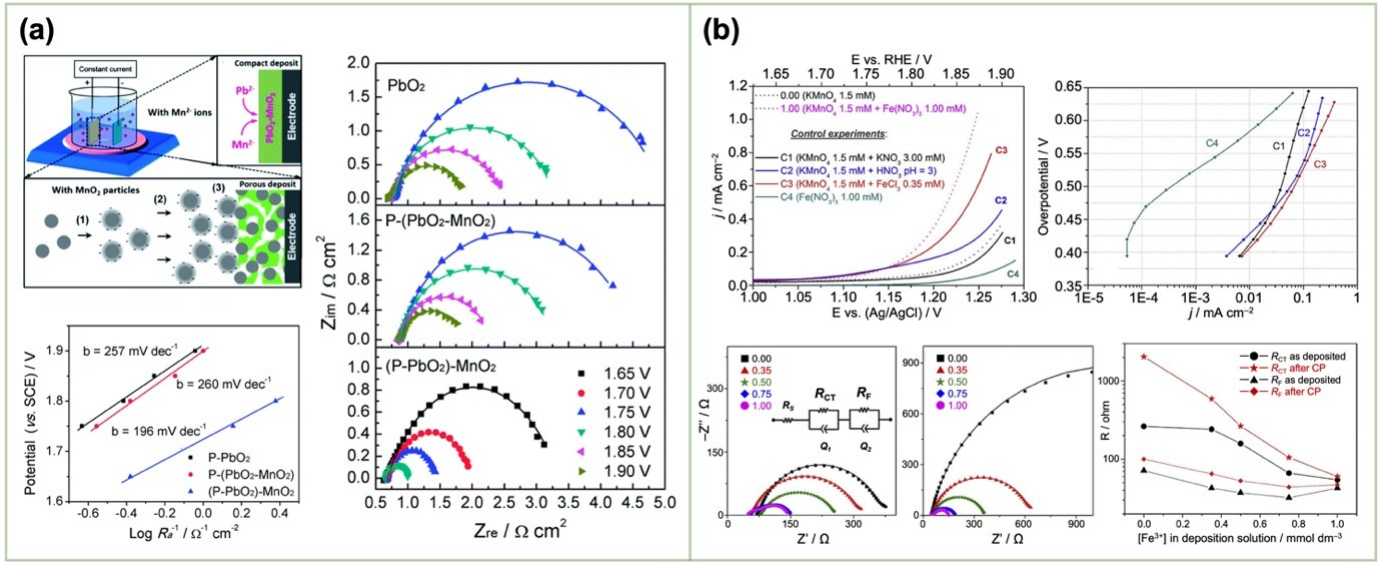
(a) Synthetic procedures for fabricating P‐(PbO_2_–MnO_2_) and (P‐PbO_2_)–MnO_2_ as well as their Tafel and Nyquist plots in acidic electrolytes compared with those of PbO_2_. Reproduced with permission from Ref.^[120]^ Copyright 2014, Royal Society of Chemistry. (b) Voltammetry and electrochemical impedance (EIS) analysis of the OER process using the electrodeposited Fe‐MnO_x_ films on the FTO substrate in a Na–Pi buffer (pH 7). The tested films were prepared at different Fe(NO_3_) concentrations. Reproduced with permission from Ref.^[121]^ Copyright 2014, Royal Society of Chemistry.

Osowiecki *et al*. performed the surfactant (SDS)‐mediated electrodeposition of MnO_2_ from a bath containing Mn(OAc)_2_ + SDS via potential cycling between 2.15 and −0.75 V vs. SHE. The resulting nanostructured MnO_2_ exhibited a current density of 0.5 mA cm^−2^ with an overpotential that was as low as 500 mV.^[49]^ A variety of nanostructured MnO_x_ films were prepared on an FTO substrate via anodic deposition at 200 μA cm^−2^ in a hydrated ionic liquid (ethylammonium nitrate) at 120 °C. The films comprising the δ‐MnO_2_‐like phase and Mn_2_O_3_ demonstrated enhanced catalytic activities in water oxidation. The catalytic activities were affected by the more open structure of the deposits.^[119]^ Li *et al*. proposed a facile method for fabricating (P‐doped PbO_2_)–MnO_2_ bicontinuous catalyst on a Pb substrate^[120]^ based on the anodic deposition of PbO_2_ in a pyrophosphate solution containing MnO_2_ particles (Figure 11a). For comparison, P‐doped (PbO_2_–MnO_2_) was anodically co‐deposited by adding Mn^2+^ to the pyrophosphate solution. The anode comprising P‐(PbO_2_–MnO_2_) with a MnO_2_ content of 0.60 wt % exhibited marginally enhanced activity compared with the P‐PbO_2_ anode. Regarding the (P‐PbO_2_)–MnO_2_ catalyst with a MnO_2_ content of 8.17 wt %, the calculated overpotential for OER is 139 mV lower than that of the P‐PbO_2_ electrode. Additionally, the calculated Tafel slopes of the P‐PbO_2_, P‐(PbO_2_–MnO_2_), and (P‐PbO_2_)–MnO_2_ anodes are 257, 260, and 196 mV dec^−1^, respectively. The enhanced electrocatalytic activities are attributed to the incorporation of MnO_2_ catalysts, which significantly reduce the adsorption resistance of *–OH_ads_, as well as the well‐defined bicontinuous structure, which facilitates ion and electron transport and contacts.^[120]^ Etzi Coller Pascuzzi *et al*. electrodeposited Fe‐MnO_x_ films onto an FTO substrate and deployed them as catalysts for OER in a 0.1 M Na–Pi buffer (pH 7).^[121]^ The proposed protocol comprised galvanostatic electrolysis at 0.25 mA cm^−2^ in 1.5 mM KMnO_4_ solutions containing different concentrations (0–1.25 mM) of Fe(NO_3_)_3_. The Tafel slope decreased drastically from 270–103 mV dec^−1^ in the presence of Fe. Moreover, the incorporation of Fe^3+^ within the electrodeposited film exerted a limited effect on the final Fe‐MnO_x_ structure. This resulted in a defective δ‐MnO_2_‐type structure with a significant fraction of surface‐Mn^3+^ species (Figure 11b). The resistance of the catalyst layer decreased with the increasing concentration of Fe^3+^ in the deposition bath.

**Figure 12:**
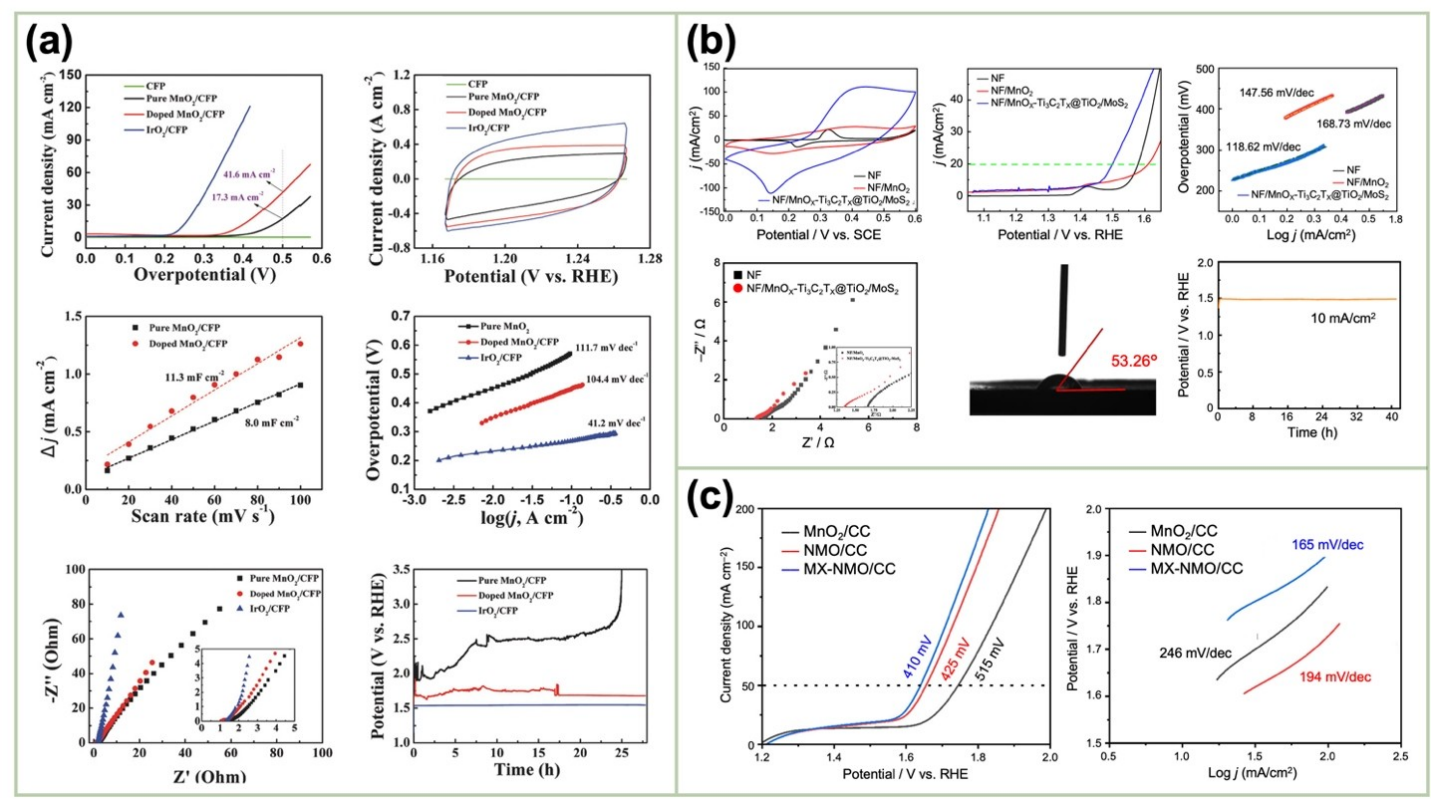
OER characteristics measured in a 1 M KOH solution. (a) CFP‐supported metal‐ion (Fe, V, Ci, and Ni)‐doped MnO_2_ ultrathin NSs. Reproduced with permission from Ref.^[122]^ Copyright 2017, Wiley‐VCH. (b) MnO_x_–Ti_3_C_2_T_x_@TiO_2_/MoS_2_ supported on NF. Reproduced with permission from Ref.^[124]^ Copyright 2021, Springer Nature. (c) CC‐supported MX–NMO. Reproduced with permission from Ref.^[125]^ Copyright 2022, American Chemical Society.

Ye *et al*. fabricated MnO_2_ ultrathin NSs doped with multiple metals (Fe, V, Co, and Ni) on carbon fiber paper (CFP) using a facile anodic co‐deposition method from a mixed electrolyte of 0.20 M MnSO_4_+0.1 M NH_4_Fe(SO_4_)_2_+0.01 M NaVO_3_+0.05 M Co(NO_3_)_2_ +0.01 M NiSO_4_ +1.1 M H_2_SO_4_ at 98 °C.^[122]^ As shown in Figure 12a, the doped MnO_2_ ultrathin NS/CFP and IrO_2_/CFP composite electrodes for OER achieved low overpotentials of 390 and 245 mV to deliver current densities of 10 mA cm^−2^ in 1 M KOH, respectively. The metal ions (Fe, V, Co, and Ni) were doped into pristine MnO_2_, increasing its electrochemically available surface area and decreasing its charge‐transfer resistance. The potential of the doped composite electrode for long‐term OER at a constant current density of 20 mA cm^−2^ was significantly lower than that of the pristine MnO_2_ composite electrode. Mooni *et al*. fabricated GO‐modified MnO_2_–NiO composite nanoflakes on an SS substrate. The SS electrode was anodically polarized in a deposition bath comprising Mn(OAc)_2_ and Ni(OAc)_2_ in conjunction with H_2_SO_4_ (or Na_2_SO_4_) via a potential‐cycling mode.^[123]^ Thereafter, the electrode was subjected to annealing at 400 °C. Subsequently, the SS‐supported MnO_2_–NiO nanoflakes were dropcast with a GO dispersion, followed by annealing at 200 °C. The electrode displayed a low overpotential (379 mV) and a small Tafel slope (47.84 mV dec^−1^) at a current density of 10 mA cm^−2^ in a KOH solution. Furthermore, the electrode displayed long‐term stability, with an operational lifetime of 28,800 s. Liu *et al*. fabricated a Ti_3_C_2_T_x_@TiO_2_/MoS_2_ composite by conglutinating these three materials.^[124]^ Ti_3_C_2_T_x_, a member of the MXenes family of 2D materials, has emerged as a promising candidate for application in energy storage, conductive fillers, and catalysis. Additionally, MoS_2_, a layered transition metal dichalcogenide, facilitates charge transfer at the interface with TiO_2_. As shown in Figure 12b, Ti_3_C_2_T_x_@TiO_2_/MoS_2_ increased the surface area of MnO_x_ exposed to the electrolyte, enhanced the Faradaic reaction, and reduced the ion‐diffusion length. The Tafel slope of the NF‐supported MnO_x_–Ti_3_C_2_T_x_@TiO_2_/MoS_2_ sample was 118.62 mV dec^−1^, which is lower than those of MnO_x_ (147.56 mV dec^−1^) and bare NF (168.73 mV dec^−1^). The stability of the MnO_x_–Ti_3_C_2_T_x_@TiO_2_/MoS_2_ sample was confirmed at 10 mA cm^−2^ for 42 h, with the overpotential remaining constant throughout the test duration. A crystalline δ‐MnO₂ film deposited anodically at a potentiostatic condition (1.0 V vs. Ag/AgCl) by Fujimoto *et al*. exhibited zero OER activity, which is consistent with the findings of other research groups.^[47]^ However, when the interlayer of the δ‐MnO_2_ film was ion‐exchanged with Co^2+^ instead of K^+^, it exhibited high OER activity. The observed OER activity was attributed to the Co^2+^ isolated between the MnO_2_ layers, which exhibited a mass activity (63.5 A g_‐Co_^−1^@*η*=0.4 V) that was significantly higher than that of α‐Co(OH)_2_ (33.1 A g_‐Co_^−1^@*η*=0.4 V), where Co^2+^ was bound within the hydroxide networks. Jia *et al*. reported the exceptional OER performance of MXene–Ni_0.075_Mn_0.925_O_2_ on CC prepared through a novel electrodeposition process.^[125]^ First, Ni_x_Mn_1–x_O_2_ was synthesized via anodic deposition on a CC electrode from a MnSO_4_ +Na_2_SO_4_ solution containing various concentrations of Ni(NO_3_)_2_ at a constant current of 3 mA. Subsequently, the Ni_x_Mn_1–x_O_2_ electrode was subjected to an electric field of 5 V in an MXene‐containing solution to facilitate MXene deposition. The incorporation of MXene into the electrode structure substantially increased the surface area and generated electron‐conduction channels, which collectively enhanced the electrochemical performance. In Figure 12c, the CC‐supported MXene–Ni_0.075_Mn_0.925_O_2_ electrode (MX–NMO/CC) exhibited an overpotential of 410 mV at a constant current density of 50 mA cm^−2^, which was approximately 105 mV smaller than that of the pristine MnO_2_/CC electrode. The MX–NMO/CC electrode exhibited a smaller Tafel slope (165 mV dec^−1^) than MnO₂/CC (194 mV dec^−1^) and NMO/CC (246 mV dec^−1^), corresponding to the fast kinetics for OER. The deposition of MXene on the surface of MnO_2_ significantly enhances its conductivity. The introduction of Ni cations resulted in the generation of additional defects and oxygen vacancies, which facilitated enhanced electron and mass transport. Therefore, the enhanced OER performance of MX–NMO/CC could be attributed to the combined effect of the metal‐like conductivity of MXene as well as the defect‐rich MnO_2_ enabled by Ni‐ion doping.

#### 5.1.3 Seawater Electrolysis

**Figure 13:**
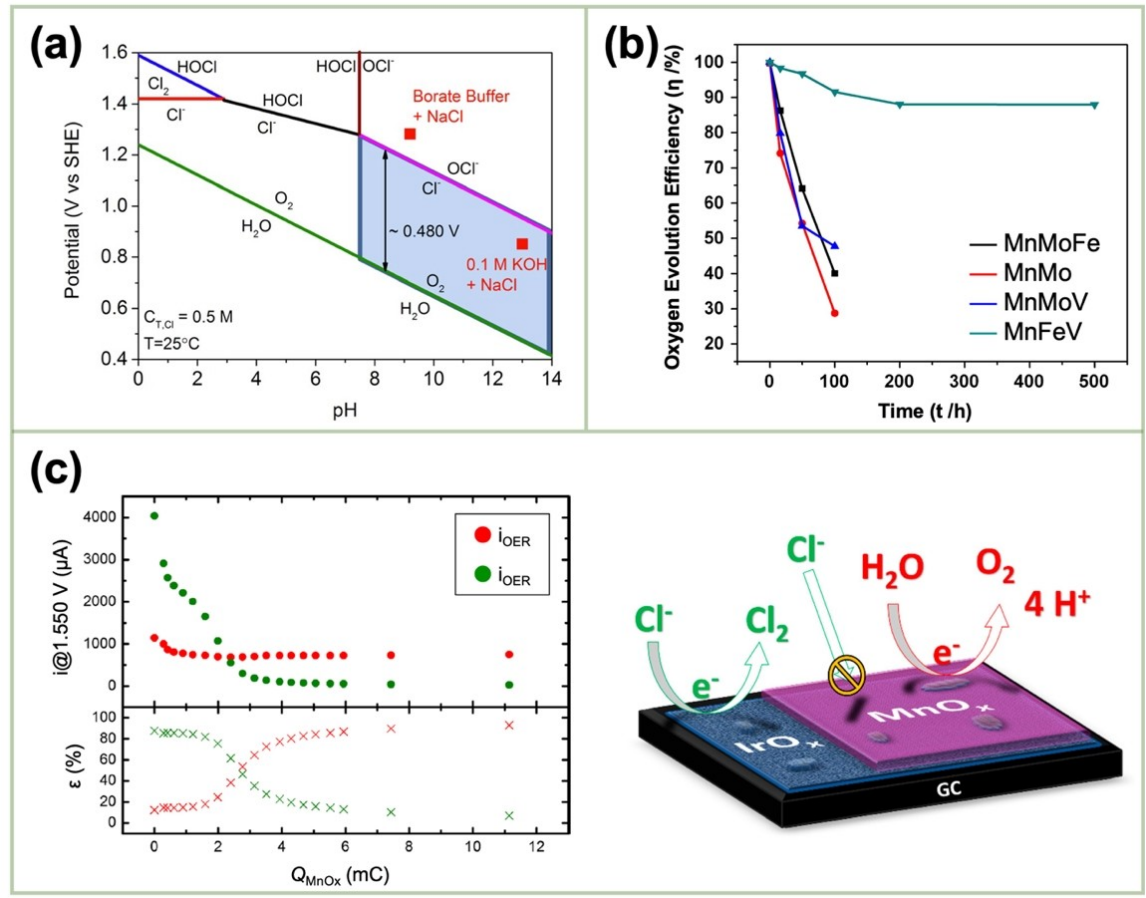
(a) Pourbaix diagram of an artificial seawater model: dissolved 0.5 M NaCl solution with no other Cl sources. Reproduced with permission from Ref.^[122]^ Copyright 2016, Wiley‐VCH. (b) Time dependence of OER in the electrolysis of a NaCl solution using the noted catalysts. Reproduced with permission from Ref.^[136]^ Copyright 2012, Elsevier. (c) OER/COR selectivity as a function of the thickness of the MnO_x_ (*Q*_MnOx_) film, along with a sketch for the origin of the selectivity. Reproduced with permission from Ref.^[137]^ Copyright 2018, American Chemical Society.

The development of OER catalysts as well as the introduction of green hydrogen production via large‐scale electric water splitting may eventually result in the depletion of fresh water, similar to the depletion of fossil fuels.^[129]^ However, there is considerable interest in resolving this concern through seawater electrolysis for hydrogen production. Seawater, which constitutes 97 % of all water on Earth, represents a significant source of hydrogen production.^[130]^ The strategy employed for seawater electrolysis to enhance the kinetics of anodic OER, which represents a rate‐limiting step in water splitting, is analogous to the aforementioned OER in freshwater. However, in seawater containing elevated Cl^−^ concentrations, the chloride‐oxidation reaction (COR) occurs (Equations (26) and (27)) and competes with OER. The generated oxygenated chlorine species are highly corrosive and toxic, presenting a significant challenge to seawater electrolysis. Thus, suppressing COR and promoting OER are necessary.^[130]^ Although OER is thermodynamically more favorable than COR (Figure 13a),^[131]^ OER involving four‐electron transfer is kinetically less favorable than COR involving two‐electron transfer. Thus, COR represents the dominant reaction in the electrolysis of ordinary sodium chloride (NaCl) aqueous solution using representative OER catalysts, such as IrO_2_ and RuO_2_.^[132]^ This process is not simply about increasing the catalyst activity; rather, it requires the improvement of its selectivity toward OER. It has been postulated for over two decades that electrodeposited Mn oxides favor chlorine‐free seawater electrolysis.

In an acidic electrolyte:
(26)

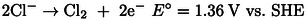




In an alkaline electrolyte:
(27)

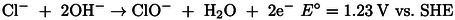




Hashimoto *et al*. introduced a series of transition metal‐doped MnO_x_/IrO_2_/Ti anodes exhibiting remarkable OER selectivity (>90 %) and stability for the electrolysis of a 0.5 M NaCl solution at pH 12.[^133^, ^134^, ^135^] Among the tested electrodes, the anodically deposited Mn–Mo electrode exhibited 100 % OER selectivity at an applied current density of 100 mA cm^−2^. The most effective catalyst was produced on an IrO_2_‐coated Ti substrate at a constant current density of 60 mA cm^−2^ in a 0.2 M MnSO_4_ +0.003 M Na_2_MoO_4_ solution at 90 °C. Similarly, Jiang *et al*. demonstrated a Mn–Fe–V anode with a Faradaic efficiency of 87.96 % for OER for 500 h (Figure 13b).^[136]^ The catalyst was anodically prepared from a 0.2 M MnSO_4_ +0.01 M Fe(NH_4_)_2_(SO_4_)_2_ +0.01 M NaVO_3_ solution at 90 °C by applying a constant current density of 60 mA cm^−2^. Further tests revealed that MnO_x_ was not a catalytically active phase, rather functioning as a water‐permeation layer and Cl^−^‐diffusion barrier (Figure 13c). For instance, Vos *et al*. deposited a MnO_x_ film on GC‐supported IrO_2_, notably decreasing the COR selectivity from 86 % to <7 % while significantly enhancing the OER selectivity.^[137]^ Conversely, Abe *et al*. identified a MnO_x_ catalyst exhibiting catalytic activity and selectivity toward OER.^[138]^ They introduced layer disorder as well as oxygen vacancies by subjecting anodically deposited Na‐intercalated layered MnO_2_ (δ‐MnO_2_) to heat treatment. The developed catalyst exhibited an OER efficiency of 87 % in a 10 mA^−2^ electrolysis of 0.5 M NaCl solution, demonstrating its potential for practical applications. As described in Section 5.1.1, the heat treatment of δ‐MnO_2_ facilitates OER activity,[^115^, ^116^, ^117^] but this may have been specific to OER and not COR.

### 5.2 Hydrogen Evolution Reaction

The integration of a HER activity into the aforementioned MnO_2_‐based catalysts exhibiting OER activity would yield bifunctional catalysts for water splitting. However, the intrinsic inactivity of MnO_2_ requires its combination with other metal elements exhibiting HER activity to achieve the desired outcome. Additionally, the introduction of foreign metal elements could regulate the electronic structure and surface properties of MnO_2_. The HER properties of the MnO_2_‐based electrodeposits are summarized in Table 4.

**Table 4:** Synthetic procedures and HER properties of reported MnO_2_‐based nanocomposites obtained by electrodeposition

Synthesis					Electrochemical test				Ref.
Deposit on substrate	Substrate	ED bath composition	Procedure for ED	Post‐treatment	Electrolyte	Scan rate	Overpotential@ 10 mA cm^−2^	Tafel slope	
Globular‐flower‐like MnO_2_/Co_3_PO_4_	NF	30 mL of PBS+50 mL of (0.15 mM MnSO_4_+0.15 mM Co(NO_3_)_2_)	Constant voltage (+1.8 V)		1 M KOH	5 mV s^−1^	102 mV	102.24 mV dec^−1^	[139]
Na‐MnO_2–x_	SS	0.16 M MnSO_4_+0.16 M Na_2_SO_4_	Constant voltage (−1.4 V)	Cycled between 0 and 1.2 V in 1 M Na_2_SO_4_	1 M KOH	5 mV s^−1^	439.7 mV	161.7 mV dec^−1^	[140]
Pd‐MnO_2_	TNA	0.1 M Mn(OAc)_2_ + NH_4_OAc	Constant potential (−1.6 V vs. Ag/AgCl)	‐	0.5 M H_2_SO_4_	10 mV s^−1^	63 mV		[141]

**Figure 14:**
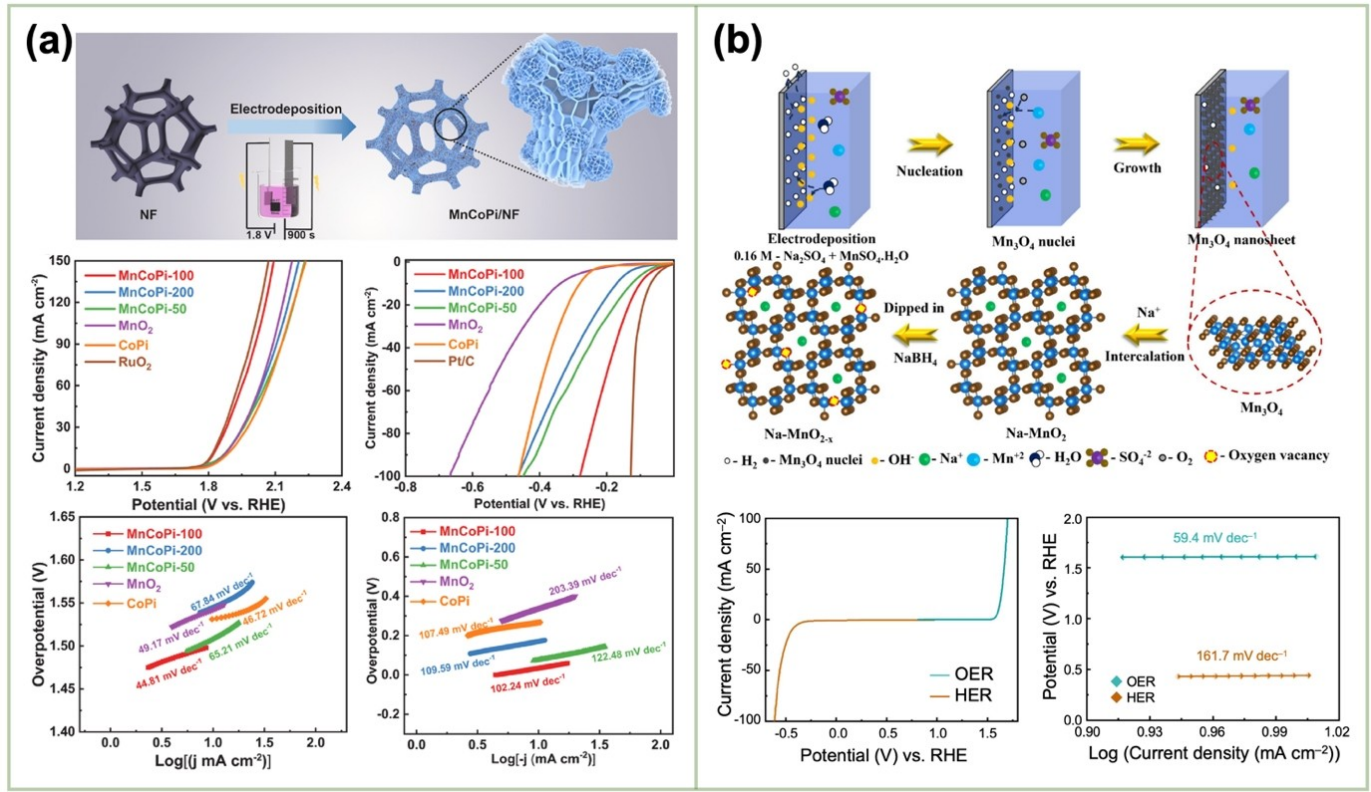
Schematic of the synthetic procedures as well as OER/HER characteristics. (a) NF‐supported MnCoPi, MnO_2_, and CoPi catalysts in a 1 M KOH solution. Reproduced with permission from Ref.^[139]^ Copyright 2024, Elsevier. (b) Oxygen‐vacancy‐enriched Na‐MnO_2–x_ film electrode in a 1 M Na_2_SO_4_ solution. Reproduced with permission from Ref.^[140]^ Copyright 2024, Elsevier.

Yang *et al*. recently synthesized a novel globular‐flower‐like MnO_2_/Co_3_(PO_4_)_2_ (denoted as MnCoPi) electrocatalyst on NF through a facile one‐step electrodeposition route from a PBS solution containing MnSO_4_ and Co(NO_3_)_2_ by applying a constant potential of +1.0 V (Figure 14a).^[139]^ The MnCoPi electrocatalyst exhibited remarkable electrocatalytic activities in a 1 M KOH solution for OER and HER. The prepared composite with a molar ratio of 100 : 1 (denoted as MnCoPi‐100) exhibited low overpotentials of 225 and 102 mV for OER and HER, respectively, delivering a current density of 10 mA cm^−2^. In a further development, Thanigai Vetrikarasan *et al*. synthesized oxygen‐vacancy‐enriched Na‐MnO_2–x_ using a simple, scalable, and inexpensive electrodeposition method.^[140]^ As illustrated in Figure 14b, the oxygen vacancies were introduced by dipping the electrodeposited film into a sodium borohydride solution. The enrichment of oxygen vacancies effectively enhanced the conductivity and reaction kinetics of the Na‐MnO_2_ electrode. The Na‐MnO_2–x_ film electrode exhibited a high specific capacitance of 395 F g^−1^ at a scan rate of 5 mV s^−1^, with a high capacitance retention of 85.9 % after 10,000 cycles. Furthermore, the Na‐MnO_2–x_ films exhibited overpotentials of 439.7 and 381.2 mV for HER and OER, respectively, generating a current density of 10 mA cm^−2^. The estimated Tafel slopes of the HER and OER of the Na‐MnO_2–x_ film were 161.7 and 59.4 mV dec^−1^, respectively. Thus, the electrodeposited, oxygen‐vacancy‐enriched Na‐MnO_2–x_ film electrode may exhibit sufficiently significant potential for application in flexible energy storage and electrocatalysis. Qiang Wu *et al*. employed TNAs as a support matrix to fabricate a Pd‐MnO_2_/TNAs composite via successive electrodepositions.^[141]^ The initial electrodeposition was performed at a constant potential of −1.6 V vs. Ag/AgCl in a solution comprising Mn(OAc)_2_ and NH_4_OAc. Pd was deposited onto the MnO_2_/TNAs electrode from a Pd(NO_3_)_2_ +KCl solution by applying a constant potential of −0.75 V. Additionally, Pd‐MnO_2_/TNAs required overpotentials of 63 and 92 mV for HER in a 0.5 M H_2_SO_4_ solution to generate current densities of 10 and 20 mA cm^−2^, respectively, which were significantly lower than those (225 and 287 mV) observed for MnO_2_/TNA. Pd‐MnO_2_/TNA exhibited the lowest Tafel slope (120 mV dec^−1^), indicating the most rapid electrochemical kinetics.

### 5.3 Oxygen Reduction Reaction

The increasing global energy consumption contributes to the exacerbation of two significant environmental concerns: the depletion of fossil fuels and global warming. Such environmental concerns have prompted significant exploration of high efficiency, environmentally friendly alternative energy‐storage and ‐conversion technologies. Among the wide variety of electrochemical energy conversion devices, FCs are exceptional as exemplars of efficiency, sustainability, and renewability. Their high safety, superior energy‐conversion efficiency, facile operation, and minimal greenhouse gas emissions distinguish them as a paragon of environmentally conscious technology. However, the existing bottleneck of FC commercialization comprises their slow cathodic ORR rate, which will result in the instability and low energy conversion efficiency of FC devices. The utilization of precious metal‐based catalysts, such as Pt, Pd, Au, and Ag can overcome the low ORR rate and has been widely explored to improve the reaction rate. However, these catalysts are expensive and rare; moreover, their low stability represents a significant barrier to their large‐scale commercialization. ORR represents the reverse reaction of OER, and the intermediates are identical in both reactions, i. e., *OH, *O, and *OOH. ORR proceeds in the reverse direction of Equations (18)‐(21) in an alkaline electrolyte, while in an acid, it proceeds in the way through Equations (25)–(22).

In the development of non‐precious metal catalysts, transition metal/metal oxides (Mn, Co, Ni, etc.) and their composites have been considered candidates for ORR catalysts owing to their diverse chemical compositions, as well as their valence states and crystal structures. Particularly, MnO_2_ represents a highly promising catalyst for ORR, exhibiting high electrocatalytic activity, environmental compatibility, and natural abundance. The catalytic performance of MnO_2_ is contingent upon its crystallographic structure and morphology. According to Cheng *et al*., the catalytic activity follows the order: α‐>β‐>γ‐MnO₂ while excluding the morphological influence.^[142]^ However, the intrinsic properties of MnO₂ are not sufficient for deployment in other applications, as it is a poor conductor. This section presents a review of ORR electrocatalysts that include the electrodeposition of Mn oxides in their fabrication process; their synthetic procedures and performances are summarized in Table 5.

**Table 5:** Synthetic procedures and ORR properties of reported electrodeposition‐prepared MnO_2_‐based nanocomposites.^[a]^

Synthesis					Electrochemical test					Ref.
Deposit on Substrate	Substrate	ED bath composition	ED Procedure	Post‐treatment	Test electrolyte (O_2_‐saturated)	Test condition	Potential (vs. RHE)	Tafel slopes	*n* value from the *K–L* plot	
P‐NS‐MnO_2_@Mn	GC coatedwith Cu sheet	0.18 M MnSO_4_+0.9 M (NH_4_)_2_SO_4_+90 mg L^−1^ SeO_2_	Constant current (500 mA cm^−2^)		0.1 M KOH	5 mV s^−1^ 1,600 rpm	*E*_1/2_=0.86 V	49.65 mV dec^−1^	3.85–4.01	[143]
MnO_2_/rGOnovel yarn‐rod	GC coated with GO	5 mM MnSO_4_ + 0.1 M Na_2_SO_4_	Potential cycling (0.4–1.3 V vs. Ag/AgCl)		0.1 M KOH	5 mV s^−1^ 1,600 rpm	*E*_onset_=0.83 V		3.4	[144]
MnO_2_/SnO_2_@NC	GC coated SnO_2_@NC	0.01 M KMnO_4_+0.01 M H_2_SO_4_	Potential cycling (0.3–−0.5 V vs. Ag/AgCl)		0.1 M KOH	50 mV s^−1^ 1,600 rpm	*E*_1/2_=0.878 V (0.879 V for Pt/C)	120 mV dec^−1^ (121 mV dec^−1^ for Pt/C)	3.92 (3.97 for Pt/C)	[145]
rGO/MnO_2_, rod‐like γ‐MnO_2_	NF	1.5 g L^−1^ MnO_2_ +1.5 g L^−1^ GO (GO was reduced to rGO)	EPD Constant voltage (2–4 V)		0.1 M KOH	1,600 rpm			2.4	[86]
α‐MnO_2_	GC with super‐aligned electrospun carbon nanofibers (ECNFs)	0.01 M MnSO_4_+0.1 M Na_2_SO_4_	Constant current (45 μA)		20 mM KCl				3.84	[146]
rGO/MnO_2_/Ag	GC coated with rGO	0.01 M KMnO_4_+0.01 M H_2_SO_4_	Potential cycling (1.3–0.5 V vs. Ag/AgCl)	Potential cycling in Ag‐containing solution	0.1 M KOH	10 mV s^−1^	*E*_onset_=0.9 V (1.1 V for Pt/C)	120.2 mV dec^−1^ (118.3 mV dec^−1^ for Pt/C)	3.9 (3.85 for Pt/C)	[147]
CMT@N‐RGO /MnO_2_	NF coated with CMT@N‐RGO	0.15 M Mn(OAc)_2_+0.15 M Na_2_SO_4_	Constant voltage of 2–8 V		0.1 M KOH	1,600 rpm				[148]

[a] P‐NS: porous nanosheet; NC: nitrogen‐doped CNTs; CMT: carbon microtubes.

**Figure 15:**
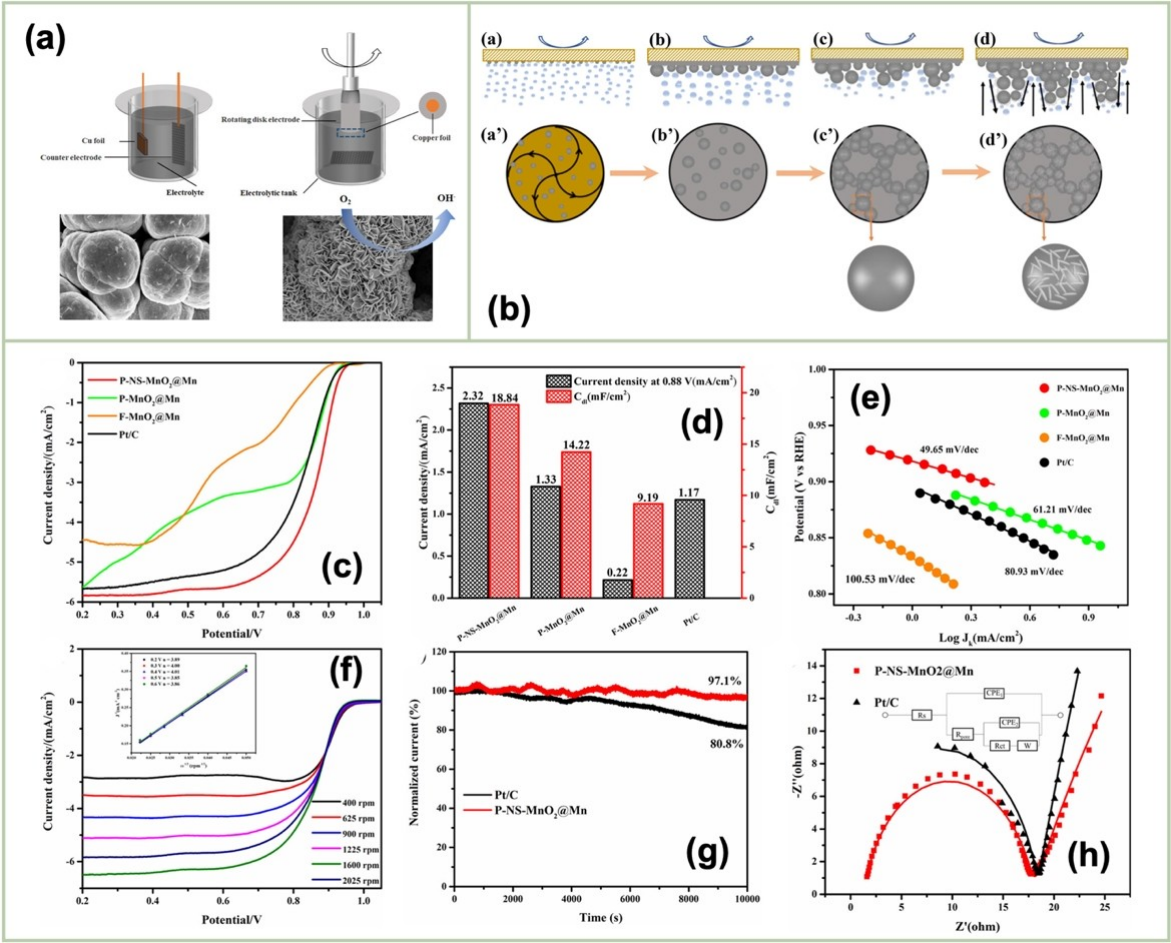
(a, b) Schematic of the synthetic procedure and (c–h) ORR characteristics of the noted catalysts in a KOH solution. (c, d) Linear sweep voltammetry analysis, (e) Tafel plots, (f) *K–L* plots, (g) chronoamperometry curves, and (h) EIS spectra. Reproduced with permission from Ref.^[143]^ Copyright 2024, American Chemical Society.

Meng *et al*. presented a 3D porous superimposed NS catalyst composed of a metal Mn (matrix) covered with MnO_2_ nanofilms (P‐NS‐MnO_2_@Mn) as a promising ORR catalyst.^[143]^ The 3D porous structure was embedded using a rotating disk electrode (RDE) (Figures 15a and b). During galvanostatic polarization at 500 mA cm^−2^ in a 0.18 M MnSO_4_ +0.90 M (NH_4_)_2_SO_4_ +90 mg L^−1^ SeO_2_ solution, the continuous evolution of H_2_ bubbles acted as a template that promoted the formation of a porous structure on the electrodeposited metal Mn. The cathodically formed 3D porous Mn was coated with MnO_2_. The Mn‐core/MnO_2_‐shell structure ensured rapid electronic transport between the active catalytic sites and metal‐Mn support (Figure 15g). The P‐NS‐MnO_2_@Mn catalyst exhibited a half‐wave potential (*E*_1/2_) of 0.86 V, which was higher than those of the porous (P)‐MnO_2_@Mn (0.83 V), flat film (F)‐MnO_2_@Mn (0.71 V), and commercial Pt/C (0.83 V) catalysts (Figures 15b and c). Furthermore, no significant decay was observed for a minimum of 30 h during the galvanostatic test (Figure 15f). The Tafel slope of P‐NS‐MnO_2_@Mn was 49.65 mV dec^−1^, which was significantly lower than that of the commercial Pt/C (80.93 mV dec^−1^; Figure 15d). The *K–L* plots demonstrate a linear relationship over the potential region of 0.6–0.2 V. The calculated electron‐transfer number (*n*) using the *K–L* plots in Figure 15e was within the 3.85–4.01 range, indicating that the ORR on P‐NS‐MnO_2_@Mn proceeded through a four‐electron transfer pathway.

**Figure 16:**
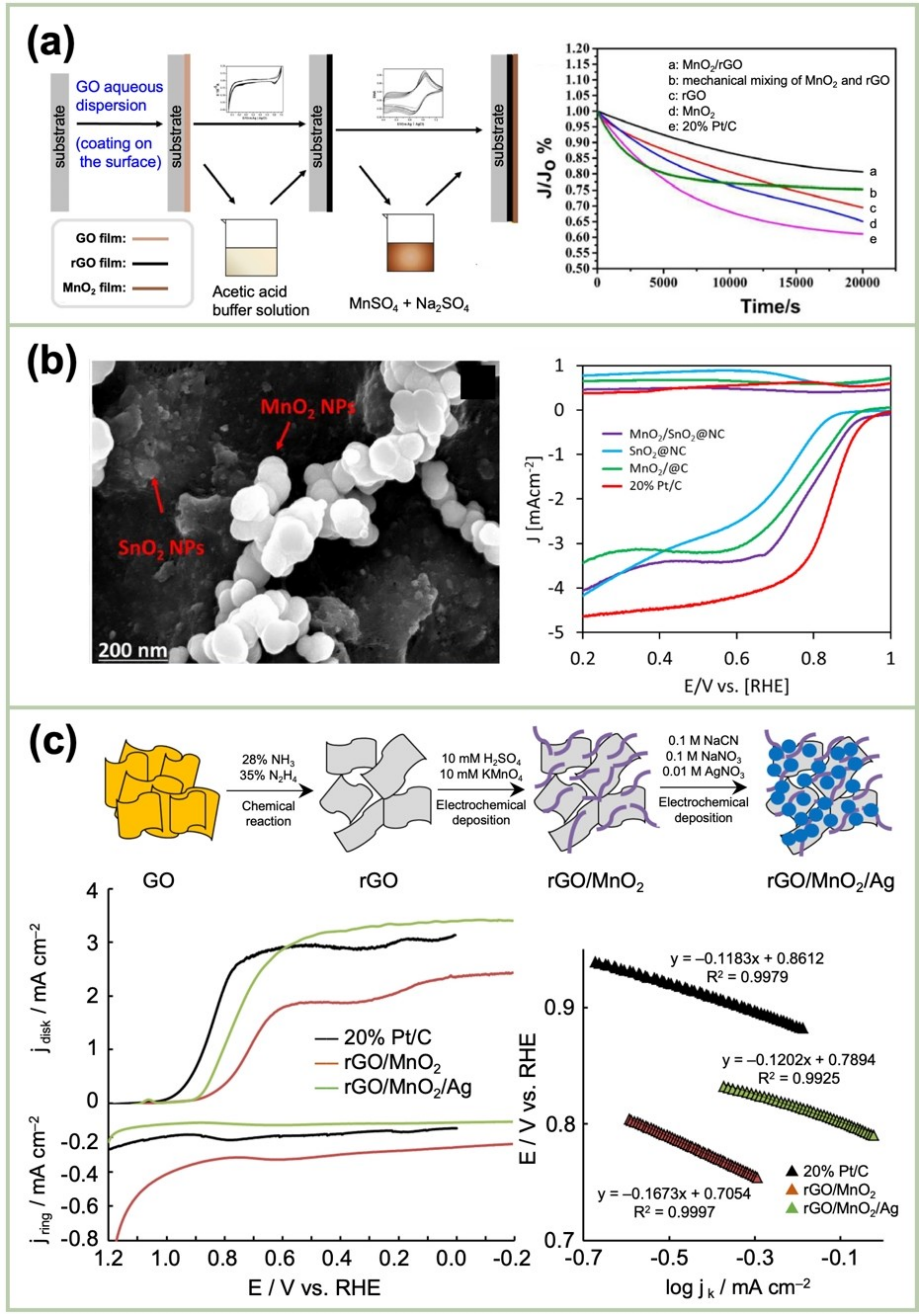
ORR characteristics of the noted catalysts measured in 1.0 M KOH. (a) MnO_2_/rGO on a GC substrate with its synthetic procedure. Reproduced with permission from Ref.^[144]^ Copyright 2017, Wiley‐VCH. (b) MnO_2_/SnO_2_/NC on a GC substrate. Reproduced with permission from Ref.^[145]^ Copyright 2021, Springer Nature. (c) rGO/MnO_2_/Ag on a GC substrate. Reproduced with permission from Ref.^[146]^ Copyright 2022, American Chemical Society.

**Figure 17:**
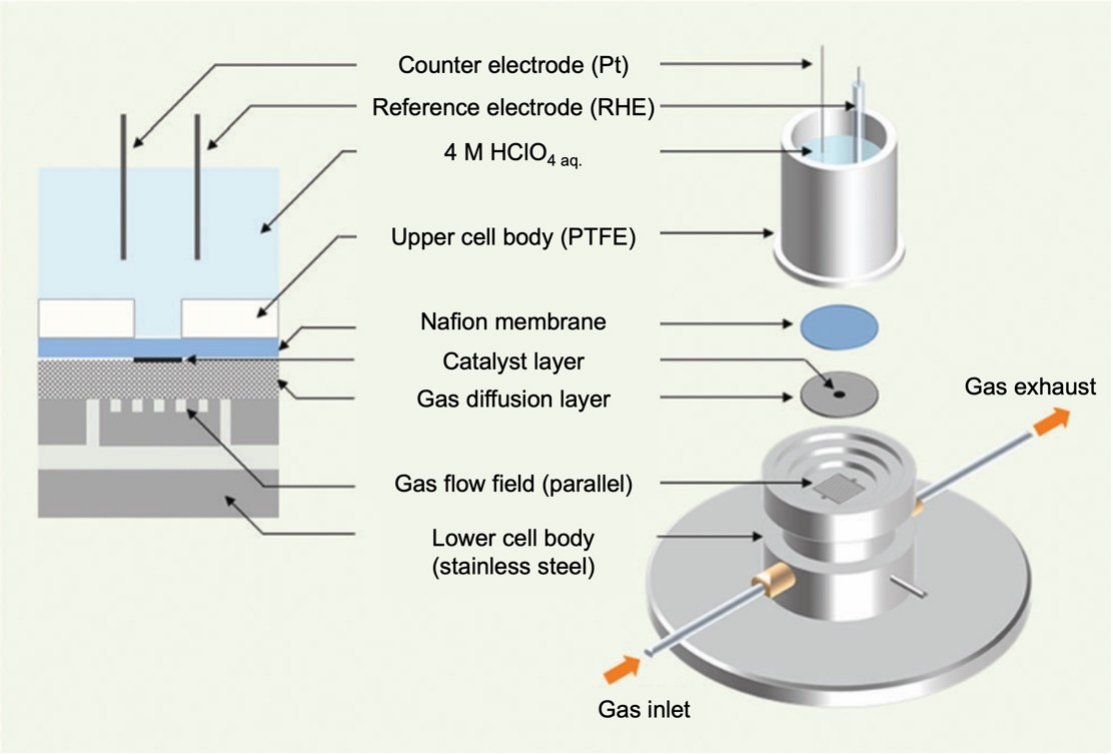
Schematic of the GDE half‐cell. Reproduced with permission from Ref.^[150]^ Copyright 2018, Royal Society of Chemistry.

Huang *et al*. fabricated a MnO_2_/rGO composite on a GC substrate exhibiting a novel yarn‐rod shape via electrodeposition.^[144]^ As shown in Figure 16a, the first step involved coating a GO solution directly onto the surface of the GC substrate, which was subsequently reduced electrochemically. The obtained rGO/CC electrode was subjected to anodic potential cycling between 0.4 and 1.3 V vs. Ag/AgCl in a MnSO_4_ +Na₂SO_4_ solution to deposit MnO_2_ on its surface. The GC‐supported MnO_2_/rGO electrode exhibited superior activity for ORR in the 1 M KOH solution compared with MnO_2_ and rGO. Furthermore, it exhibited better ORR stability, higher *n*, and stronger methanol‐tolerant ability than the commercial Pt/C catalyst. The combination of highly active tin(IV) oxide (SnO_2_) NPs on N‐induced CNT (SnO_2_@NC) by electrodeposition on GC‐RDE using electroactive MnO_2_ yielded an effective nanostructured catalyst for ORR.^[145]^ Cathodic electrodeposition was performed via potential cycling between 0.3 and 0.5 V in a KMnO_4_ +H_2_SO_4_ solution utilizing the SnO_2_/NC supported on GCE. Figure 16b shows the RDE voltammograms of the MnO_2_/SnO_2_@NC, SnO_2_@NC, MnO_2_@C, and 20 % Pt/C catalysts at a rotation speed of 1,600 rpm in an oxygen‐saturated 0.1 M KOH solution. The MnO_2_/SnO_2_@NC catalyst exhibited a higher diffusion‐limiting current density (−4.14 mA cm^−2^)) than the commercial Pt/C electrodes (−4.63 mA cm^−2^), whereas the MnO_2_@C catalyst demonstrated a relatively low diffusion‐limiting current density (−3.41 mA cm^−2^). Purwaningsih *et al*. synthesized a rGO/MnO_2_ nanocomposite on an NF substrate using a two‐step EPD technique.^[86]^ The NF electrode was submerged in a GO NS suspension at 1.5 g L^−1^. The GO particles were deposited at the anode by applying a constant voltage. Subsequently, a MnO_2_ layer was electrophoretically deposited on the GO/NF electrode from a suspension of 1.5 g L^−1^ γ‐MnO_2_, which had been chemically synthesized from MnO_4_^−^. During EPD, the GO on NF was simultaneously reduced electrochemically into rGO. The resulting rGO/MnO_2_ composite exhibited excellent electrocatalytic activity toward ORR, following a two‐electron‐transfer mechanism. Zeng *et al*. employed super‐aligned ECNFs as scaffolds for the electrodeposition of uniform Na^+^‐induced α‐type MnO_2_ from a MnSO_4_ +Na_2_SO_4_ solution at a low constant current of 45 μA.^[146]^ The data of the electrocatalytic performance revealed that the α‐MnO_2_/ECNF–GC electrode exhibited a 3.84‐electron‐transfer pathway, which was attributed to the rapid decomposition of hydrogen peroxide (H_2_O_2_) at the α‐MnO_2_ surfaces. Put differently, the electrochemically generated H₂O₂ can be decomposed into H_2_O via DISP before escaping into the bulk α‐MnO_2_ solution. Lee *et al*. prepared a porous catalyst comprising rGO‐supported Ag and MnO_2_ (denoted as rGO/MnO_2_/Ag). The catalyst was prepared through a facile electrodeposition route and deployed as an electrocatalyst for ORR in alkaline FCs.^[147]^ First, a suspension of rGO in water was dropcast onto GCE. Subsequently, the rGO/GC electrode was subjected to potential cycling between 1.3 and 0.5 V vs. Ag/AgCl in a KMnO_4_ + H_2_SO_4_ solution to deposit MnO_2_. Additionally, Ag was deposited onto MnO_2_/rGO by subjecting the material to potential cycling between 0.3 and −0.6 V in an AgNO_3_ +NaNO_3_ +NaCN solution. The Tafel slope (120.2 mV dec^−1^) of rGO/MnO_2_/Ag, as evaluated in 0.1 M KOH, was significantly lower than that of rGO/MnO_2_ (167.3 mV dec^−1^), and approached that (118.3 mV dec^−1^) of the commercial Pt/C (Figure 16c). Xiong *et al*. fabricated an eco‐friendly hybrid carbon microtube (CMT)@nitrogen‐doped rGO (CMT@N‐rGO) as an excellent carbonaceous scaffold for loading MnO_2_ nanowalls, and they were deployed as high‐performance electrodes for supercapacitors as well as ORR catalysis.^[148]^ CMT@N‐rGO was first fabricated by combining the dipping and CVD methods. Subsequently, the obtained CMT@N‐rGO was employed as a carbonaceous scaffold to electrodeposit MnO_2_ from a Mn(OAc)_2_ +Na_2_SO_4_ solution. The 3D CMT@N‐rGO/MnO_2_ hybrid represented a promising platform for the development of high‐performance bifunctional materials as supercapacitors and ORR catalysts. Marukawa *et al*. synthesized an Ag^+^‐intercalated δ‐MnO_2_ film on CFP via electrodeposition.^[149]^ The interlayer Ag^+^ was reduced in situ into Ag^0^ metal during cathodic sweep, thereby functioning as an ORR catalyst. The mass activity of the Ag grown in that confined space was significantly higher than that of Ag NPs electrodeposited from an aqueous AgNO_3_ solution. The incorporation of CFP coated with electrodeposited MnO_2_ into a gas diffusion electrode (GDE) half‐cell (Figure 17) allows for the acquisition of data with good reproducibility, without requiring specialized expertise.^[150]^

### 5.4 Bifunctional Oxygen Evolution and Reduction Reactions

The development of high‐performance, non‐precious metal bifunctional electrocatalysts for ORR and OER represents a significant advancement in the field of rechargeable metal–air batteries and regenerative FCs. The study of non‐precious metal catalysts, including transition metal oxides (such as Mn, Co, and Fe), has been a prominent research area. Among them, MnO_2_ has attracted considerable attention owing to its excellent ORR catalytic activity, inexpensiveness, and environmental compatibility. However, the OER catalytic activity of pristine MnO_2_ is not sufficient for application as an electrocatalyst for the charging reaction of metal (Zn, Li, etc.)–air secondary batteries.

**Figure 18:**
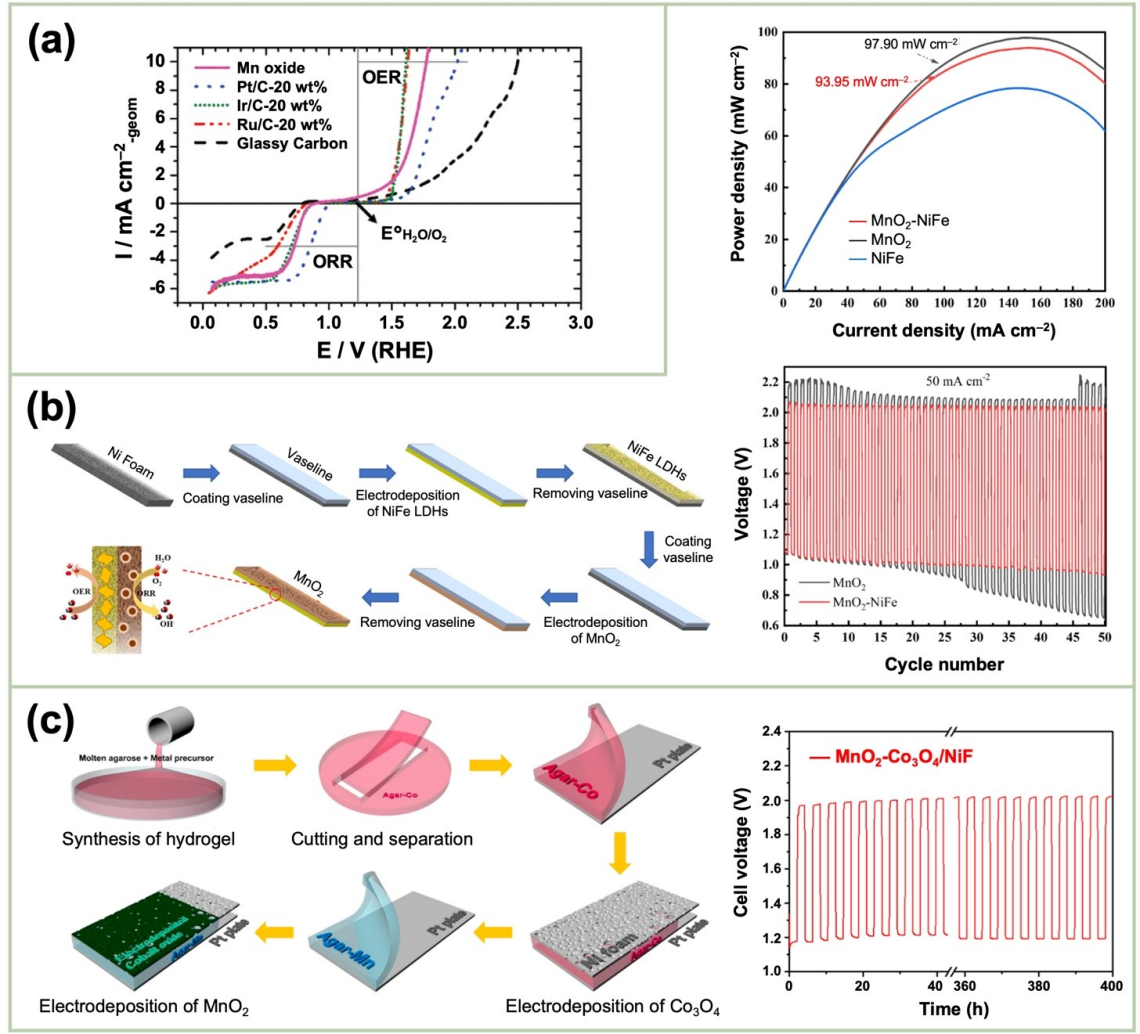
(a) Voltammograms of the MnO_x_ thin‐film‐coated GC‐RDE during anodic and cathodic scans in an O_2_‐saturated 0.1 M KOH solution. Reproduced with permission from Ref.^[6]^ Copyright 2010, American Chemical Society. (b) Schematic of the fabrication procedure for the MnO_2_–NiFe/NF electrode and the voltage profiles during current pulse and discharge curves of the zinc–air battery assembled with the aforementioned electrode compared with those of MnO_2_ and NiFe. Reproduced with permission from Ref.^[152]^ Copyright 2019, American Chemical Society. (c) Schematic of the fabrication procedure for the MnO_2_–Co_3_O_4_/NF electrode as well as the voltage profiles during current pulse for the zinc–air battery assembled with the aforementioned electrode. Reproduced with permission from Ref.^[154]^ Copyright 2016, Elsevier.

Gorlin *et al*. electrodeposited a MnO_2_ thin film on a GC RDE and subjected it to anodic and cathodic potential scans in a 0.1 M KOH solution.^[6]^ Figure 18a shows that the OER performance of the resulting pristine MnO_2_ film exhibited superior characteristics compared with Pt, and its ORR performance was superior to that of Ru. Moreover, it performed comparably with Ir. Hosseini‐Benchangi *et al*. presented a comprehensive study on the optimization of the surfactant‐assisted structure and the resultant bifunctional ORR/OER catalytic activity of anodically deposited Mn oxides on CC.^[151]^ The effects of three classes of surfactants were investigated: anionic (SDS), nonionic (t‐octylphenoxypolyethoxyethanol, Triton X‐100), and cationic (CTAB). The electrodeposition baths comprised 0.1 M Na_2_SO_4_ solutions containing Mn(OAc)_2_ and one of the surfactants at varying concentrations. Anodic polarization was conducted at 0.8, 1.2, and 1.60 V vs. Hg/HgO/20 % KOH. The utilization of CTAB promoted the formation of MnO_x_ exhibiting a nanoneedle (1D) morphology, whereas the additions of SDS and Triton X‐100 resulted in the generation of nanospherical‐ and nanopetal‐like morphologies, respectively. This was reflected in the OER/ORR activity in an O_2_‐saturated 6 M KOH solution. The observed mass activity varied up to 49 A g^−1^ (at 1,556 mV vs. RHE) and −1.36 A g^−1^ (at 656 mV vs. RHE) for OER and ORR, respectively. Wang *et al*. developed a unique electrodeposition method for combining Ni–Fe (NiFe) layered double hydroxides (LDHs) with MnO_2_ to produce a Janus electrode comprising both materials on either side.^[152]^ One side of the NF electrode was first coated with NiFe LDHs, whereas the opposite side was insulated with Vaseline. Subsequently, the uncoated side was modified with MnO_2_ via anodic deposition from a Mn(OAC)_2_ +Na_2_SO_4_ solution at a constant potential of 0.6 V vs. SCE. The MnO_2_–NiFe/Ni electrode, which was prepared by the aforementioned method, displayed superior bifunctional activity and stability for OER and ORR compared with pristine MnO_2_. This is attributable to the rational design of the Janus bifunctional configuration, which separates the OER and ORR active materials. Notably, the exposure of the ORR active sites can significantly prevent their oxidation. The zinc–air battery assembled using the Janus MnO_2_–NiFe cathode exhibited excellent cell performance, with a peak power density of 93.95 mW cm^−2^, a high energy efficiency of 52.43 % at a current density of 50 mA cm^−2^, and superior rechargeable durability even at a large current density of 50 mA cm^−2^. Hu *et al*. presented a novel approach for enhancing the catalytic activity of MnO_2_ by integrating 3D MnO_2_ with CNT/CFP without utilizing binders. They electrodeposited 3D MnO_2_ in a Mn(OAc)_2_ +Na_2_SO_4_ solution on CNT/CFP at a constant current density of 5 mA cm^−2^. The application of MnO_2_/CNTs/CFP as a cathode in lithium–air batteries delivered a discharge capacity of 8,723.5 mAh g_‐(CNTs + MnO2)_^−1^ at 100 mA g^−1^ for the as‐prepared MnO_2_/CNTs/CFP electrode. This superior electrochemical performance is attributable to the independent nanoporous structure, which offers abundant catalytic sites for ORR. Furthermore, this process is associated with reduced side reactions.^[153]^ Kim *et al*. selectively electrodeposited MnO_2_ and Co_3_O_4_ on an NF substrate using a facile agarose‐gel‐mediated strategy to fabricate a sectionalized MnO_2_–Co_3_O_4_ electrode that avoids the physical interference of the catalytic function. First, two types of agarose‐gel media containing Mn^2+^ and Co^2+^ precursor ions (Agar–Mn and Agar–Co, respectively) were prepared. Then, a piece of the Agar–Co medium was attached to one side of NF and electrolyzed at −0.4 V vs. Ag/AgCl to deposit Co_3_O_4_. Second, Agar–Mn was attached to the opposite side, followed by the electrodeposition of MnO_2_ at 1.0 V. This novel structure enhanced the ORR and OER catalytic activities compared with pristine metal‐oxide catalyst electrodes. Even after 400 h of operation (equivalent to ~100 cycles), the sectionalized MnO_2_‐Co_3_O_4_ electrode exhibited excellent cycling performance and long‐term stability in rechargeable zinc–air batteries.^[154]^

**Figure 19:**
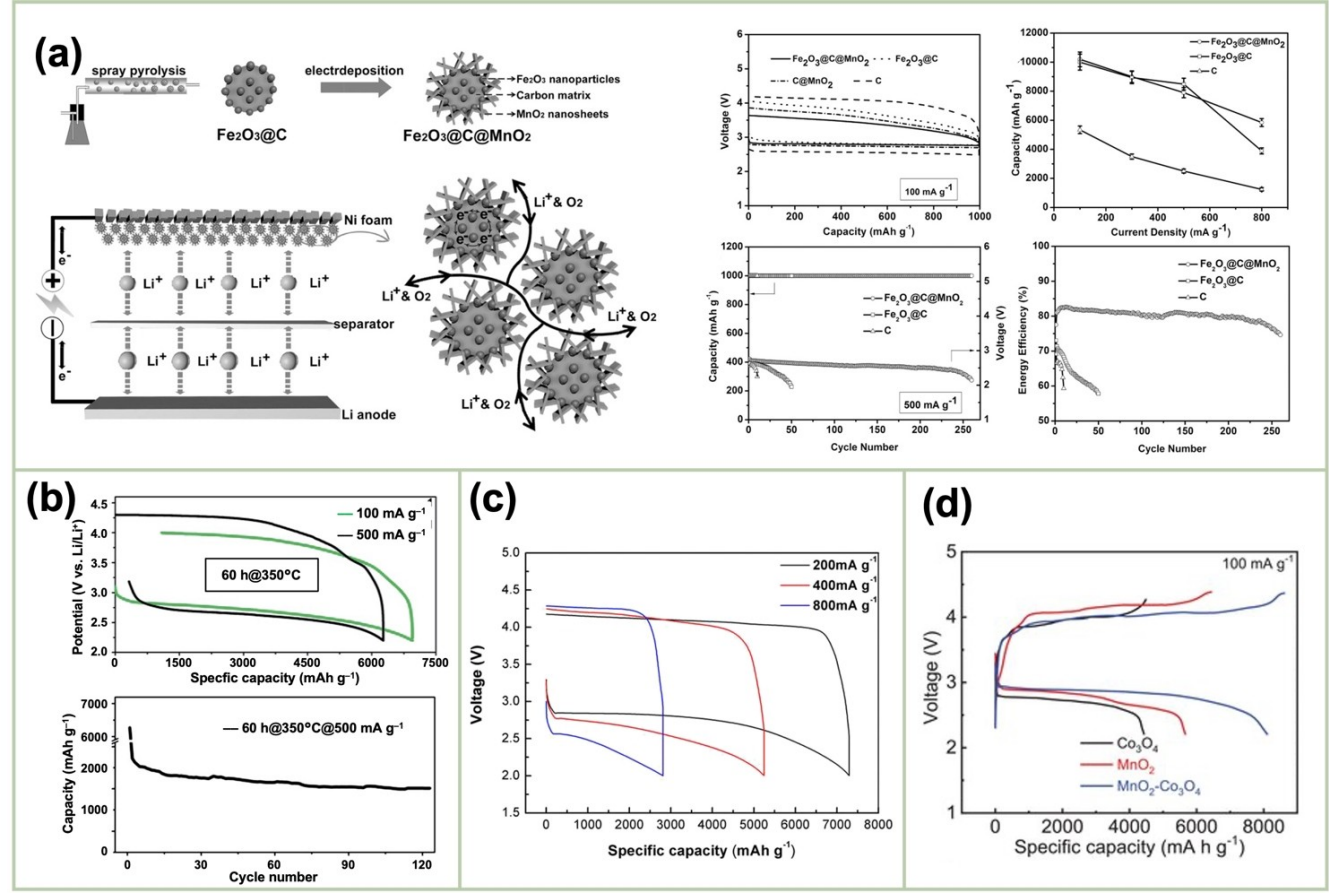
(a) Schematic of the fabrication procedure for the Fe_2_O_3_@C@MnO_2_ nanocomposite/NF electrode and the performance of the Li–air battery assembled with the aforementioned electrode compared with those assembled with MnO_2_ and Fe_2_O_3_. Reproduced with permission from Ref.^[155]^ Copyright 2015, Wiley‐VCH. (b) Performance of the Li–air battery assembled with an NF‐supported sponge‐like ε‐MnO_2_ nanostructure. Reproduced with permission from Ref.^[155]^ Copyright 2015, Wiley‐VCH. (c) Charge/discharge curves of the Li–air battery assembled with a CC‐supported MnO_2_−Co_3_O_4_@CC electrode. Reproduced with permission from Ref.^[158]^ Copyright 2023, Royal Society of Chemistry.

A novel approach for synthesizing Fe_2_O_3_@C@MnO_2_ nanocomposites via aerosol‐spray pyrolysis and electrodeposition was proposed for application in rechargeable Li–O_2_ batteries (Figure 19a).^[155]^ MnO_2_ electrodeposition was conducted galvanostatically from a Mn(OAc)_2_ +Na_2_SO_4_ solution onto NF‐supported Fe_2_O_3_@C nanocomposites at 1.5 mA cm^−2^. The superior OER/ORR bifunctional catalytic activities were attributed to the combined function of Fe_2_O_3_ and MnO_2_ in conjunction with the facile charge transfer in the carbon matrix. These activities allowed for the nanocomposite to exhibit a small overpotential (0.61 V@100 mA g^−1^), a high specific capacity (~10,200 mAh g^−1^ @100 mA g^−1^), a large energy efficiency (>80 %), and excellent stability (retaining 90 % of its initial capacity after 260 cycles at a cutoff of 1,000 mAh g^−1^ at 500 mA g^−1^) in Li–O_2_ batteries. Hu *et al*. synthesized a sponge‐like ε‐MnO_2_ nanostructure by directly growing ε‐MnO_2_ on NF through a facile electrodeposition route.^[156]^ The nanostructured ε‐MnO_2_ was anodically deposited from a Mn(OAc)_2_ +Na_2_SO_4_ solution at a constant current density of 5.0 mA cm^−2^ and annealed at 350 °C in the air. When employed as a self‐supporting binder‐free cathode material for rechargeable non‐aqueous Li–O_2_ batteries, the ε‐MnO_2_/Ni electrode demonstrated considerable high‐rate capability (discharge capacity of ~6,300 mA h g^−1^ at a current density of 500 mA g^−1^) and enhanced cyclability (exceeding 120 cycles) without controlling the discharge depth. This enhanced performance was attributed to the 3D nanoporous structure and the presence of abundant oxygen defects, as well as the absence of side reactions associated with carbon‐based conductive additives and binders. Wei *et al*. developed a cathode comprising MnO_2–x_ NS/SS that is free of carbon and binders via a facile and effective electrodeposition–solvothermal route.^[157]^ Anodic electrodeposition was conducted at a constant current density of 0.25 mA cm^−2^ in a Mn(OAc) +Na_2_SO_4_ solution. The introduction of Mn^3+^ and oxygen vacancies in MnO_2–x_ NSs notably enhanced the electronic conductivity and catalytic activity. The nanostructured MnO_2–x_/SS cathode in a non‐aqueous Li–O_2_ battery exhibited a rechargeable specific capacity of 7,300 mAh g^−1^ at 200 mA g^−1^, representing a 39 % enhancement compared with the MnO_2_/SS cathode. Furthermore, the specific capacities were 5,249 and 2,813 mAh g^−1^ at 400 and 800 mA g^−1^, respectively, which were over 30 % higher than that observed with the MnO_2_/SS cathode. Li *et al*. synthesized Co_3_O_4_ NS arrays (MnO_2_−Co_3_O_4_@CC) grafted with MnO_2_ NSs on CC for efficient bifunctional oxygen catalytic cathodes for Li−O_2_ batteries.^[158]^ First, a piece of CC was cathodically polarized at −0.8 V in a Co(NO_3_)_2_ solution, followed by annealing at 350 °C in the air. Subsequently, the Co_3_O_4_@CC electrode was subjected to anodic deposition of MnO_2_ NSs at a constant current of 0.4 mA using a Mn(OAc)_2_ +NH_4_(OAc) solution. The authors posited that the mutually perpendicular Co_3_O_4_ and MnO_2_ NSs in the grafted architecture ensured the accessibilities of the ORR and OER catalytically active sites of MnO_2_ and Co_3_O_4_, respectively, during repeated cycling. Therefore, the hybrid MnO_2_–Co_3_O_4_@CC exhibited effective bifunctional catalysis, delivering a high specific capacity of 8,115 mA h g^−1^ and a low overall overpotential of 0.64 V.

The synthetic procedures of the catalysts in this section are summarized in Table S2.

## 6 Conclusions and Perspectives

### 6.1 Electrodeposited Manganese Dioxide for Electrocatalysis

Anodic and cathodic depositions, as well as EPD, facilitate the fabrication of MnO_2_ catalyst layers on electrode substrates without requiring binders, and these substrates can be deployed as electrode devices. This has the advantage of enabling the production of a uniform catalyst layer over a larger electrode area, thereby facilitating scalability, which favors industrial‐scale commercialization. Numerous electrochemical variables are present in the electrodeposition processes, and they can effectively modulate the electronic and crystal structures as well as the morphology of the resulting MnO_2_ in a sensitive manner. Consequently, the reactivity of the catalysts can be modified. For instance, several research groups reported that MnO_2_ deposited using a potentiostatic technique exhibits zero OER activity, whereas those deposited using a potentiodynamic method display notable activity. Similarly, initially inactive films became active when electrochemically stimulated or heat‐treated, following their electrodeposition. The observed activation is associated with the presence of Mn^3+^ species in the oxide, although this has not been clarified. Although the existing results are satisfactory, further detailed analysis is required in an operand environment. Some bifunctionality (catalytic activity and large capacitance) have been reported, indicating the applicability of the same design strategy. Notably, integrating the success of supercapacitors with the development of catalysts is essential even though it will be challenging to comprehensively explain the observed bifunctionality, given that pseudocapacitance is a change in the oxidation state of Mn and cannot be directly related to a specific oxidation state (in this case, Mn^3+^), unlike in OER catalysts.

### 6.2 Electrodeposited Manganese Dioxide–Based Composites for Electrocatalysis

First, there are far fewer reports on the electrochemical synthesis of composites with other metal species compared with chemically synthesized heterometallically introduced MnO_2_. This is because of the complexity of directly electrodepositing multiple metal species during anodic deposition. However, several studies revealed the possibility of incorporating them in a heated electrodeposition bath as well as pre‐ and post‐treatments, which would improve conductivity. It is also interesting to fabricate bifunctional cathodes for metal–air batteries by electrodepositing different catalysts on sectionalized parts of the electrode. Electrodeposition is a good strategy for covering large areas with MnO_2_, as already established. However, the low conductivity and dense structure of MnO_2_ films complicate the fabrication of thick films. Fortunately, the development of supercapacitor electrodes by mixing MnO_2_ with nanocarbons has resolved this bottleneck. This method would be effective for reduction catalysts and might not be suitable for anode catalysts owing to the corrosion and dissolution of the carbon materials.

### 6.3 Toward Industrial Applications

The last decade has witnessed the commercial availability of various scaffold materials, such as CC and NF. These materials simply increase the current density per geometric area of the catalyst‐supported electrode, corresponding to increased hydrogen production in water splitting. Thus, generating the level of current density required for industrial applications might be possible. This fact would also increase the value of the electrodeposition technology. Unfortunately, MnO_2_‐based catalysts are not in the top class of transition metals for water oxidation, lagging behind Ni‐ and Co‐based catalysts in terms of geometric current densities. Therefore, to exploit their cost advantage and safety, differentiation points other than the magnitude of the current density are necessary. Particularly, in direct seawater electrolysis, which has recently attracted attention, the unique selectivity of electrodeposited MnO_2_ for oxygen evolution, which is not available in other metal‐oxide anodes, was discovered more than 20 years ago, although the mechanism is still unknown. Considering the rapid increase in the demand for hydrogen production via water electrolysis and the imminent depletion of fresh water, it is necessary to develop a technology that produces large amounts of hydrogen and oxygen without generating chlorine by supporting MnO_2_ composite materials on porous scaffold materials composed of corrosion‐resistant metals.

We believe that the recovery and reuse of electrodeposited MnO_2_ films can be achieved in a manner analogous to powdered MnO_2_, through acid dissolution followed by electrochemical deposition. This is also based on the anodic deposition technology for MnO_2_ from soluble Mn^2+^, as described in this review.

## Conflict of Interests

The authors declare no conflict of interest.

## Biographical Information

*Prof. Masaharu Nakayama is a Professor at the Graduate School of Sciences and Technology for Innovation, Yamaguchi University (Japan), and a Deputy Director for the Blue Energy Center for SGE Technology. After receiving his Ph.D. degree from Yamaguchi University, he became an associate professor at the same university in 2002 and a full professor in 2010. During this time, he also served as a visiting associate professor at the University of South Carolina (USA) and as an invited professor at the University of Nantes (France). His research focuses on the synthesis of advanced materials for energy and environmental applications*.

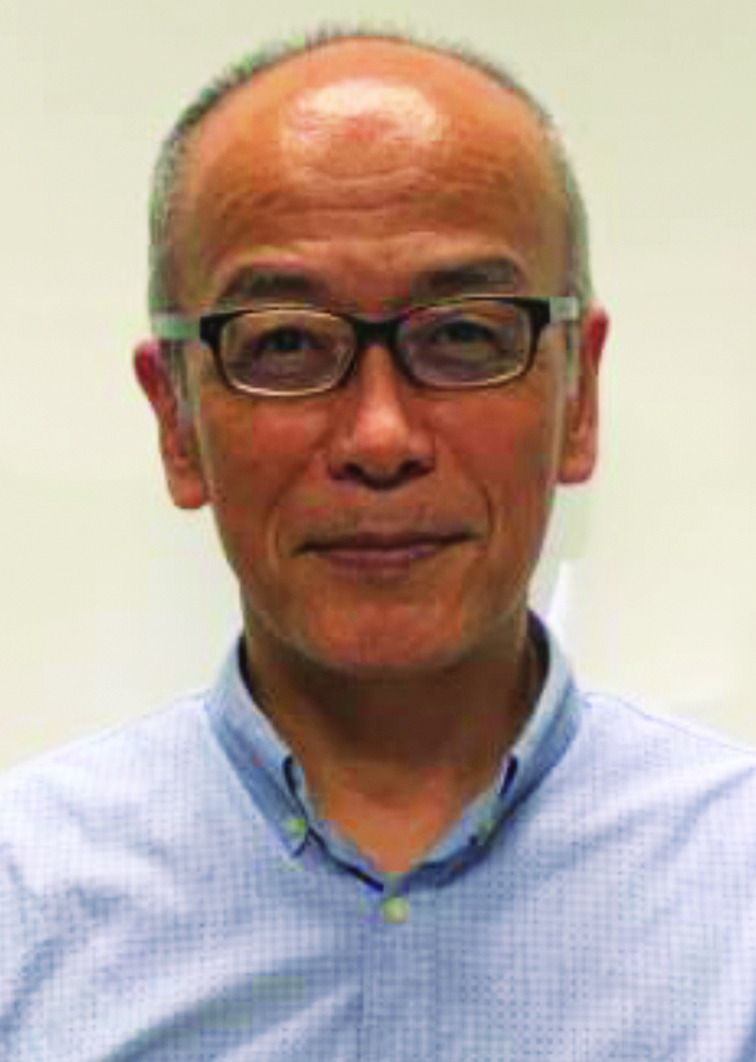

## Biographical Information

*Dr. Wataru Yoshida is an Assistant Professor at the Graduate School of Sciences and Technology for Innovation, Yamaguchi University (Japan), and a member for the Blue Energy Center for SGE Technology. He earned his Ph.D. from Kyushu University in 2020. His research initially focused on solvent extraction, ion exchange, and membrane separation. Currently, he develops catalysts for electrochemical conversion and materials for secondary battery cathodes. His work aims to improve the performance and sustainability of energy storage technologies, contributing to green energy and resource recovery advancements*.

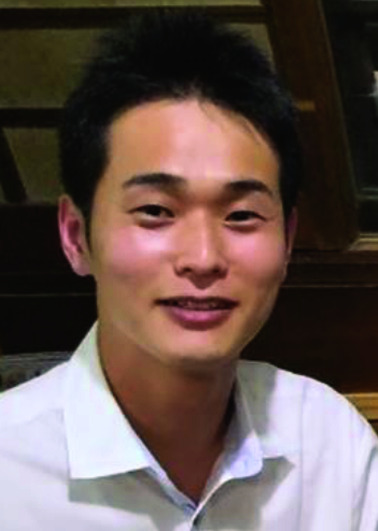

## Supporting information

As a service to our authors and readers, this journal provides supporting information supplied by the authors. Such materials are peer reviewed and may be re‐organized for online delivery, but are not copy‐edited or typeset. Technical support issues arising from supporting information (other than missing files) should be addressed to the authors.

Supporting Information
