# Supplementary material for: Electrodeposited Manganese Dioxides and Their Composites as Electrocatalysts for Energy Conversion Reactions
Source: ChemSusChem. 2024 Nov 15;18(5):e202401907. doi: 10.1002/cssc.202401907 (PMC11874673; doi:10.1002/cssc.202401907)
Supplement: Supplementary file 1 — Supporting Information [file CSSC-18-e202401907-s001.pdf]

ChemSusChem

Supporting Information

Electrodeposited Manganese Dioxides and Their Composites as Electrocatalysts for Energy Conversion Reactions

Masaharu Nakayama* and Wataru Yoshida

Supporting Information

Electrodeposited Manganese Dioxides and Their Composites as Electrocatalysts for Energy Conversion Reactions

Masaharu Nakayama[a,b,*] and Wataru Yoshida[a,b]

[a]*Department of Applied Chemistry, Graduate School of Sciences and Technology for Innovation, Yamaguchi University, 2-16-1 Tokiwadai, Ube 755-8611, Japan*

[b]*Blue Energy Center for SGE Technology (BEST), Ube 755-8611, Japan*

Table S1. Various synthetic procedures and application properties of MnO_2-based nanocomposites prepared via electrodeposition reported in the literature [a]

Synthesis						Application				Ref.
Deposit on substrate	Substrate	Bath composition<br>for ED (or EPD)	Strategy	Procedure<br>for ED (or EPD)	Post-treatment	Area	Test electrolyte	Representative performances[b]		
MnO_2 with other metals										
PtAu-MnO_2	GP paper	0.1 M Na_2SO_4<br>+ 1 mM K_2PtCl_6<br>+ 1 mM $KAuCl_4$<br>+ 50 mM $MnSO_4$	ED	potential cycling<br>(1.4– –1.5 V<br>vs. Ag/AgCl)		biosensor	0.1M PBS<br>(pH 7.4)	*sensitivity:*<br>58.54 $\mu A\ cm^{-2}\ mM^{-1}$<br>for glucose	*LOD:* 0.02 mM	[78]
MnO_2 with nanocarbons										
Graphene										
MnO_2	GP paper	0.1 M $Mn(OAc)_2$<br>+ 0.1 M Na_2SO_4	ED	potential cycling		supercapacitor	1 M KCl	328 F g^{-1}@1 mA		[81]
MnO_2	GP-coated Ni foam	20 mM $Mn(NO_3)_2$<br>+ 100 mM $NaNO_3$	ED	pulse current<br>(500 $\mu A\ cm^{-2}$)	annealed<br>at 300°C under<br>Ar atmosphere	supercapacitor	0.5 M Na_2SO_4<br>(pH 10)	1.42 F cm^{-2}@2 mV s^{-1}<br>130 F g^{-1}@2 mV s^{-1}	82% after 5000 cycles	[82]
MnO_2	3D-GP/GP fiber	1 M Na_2SO_4<br>+ 0.1 M $MnSO_4$	ED	constant current<br>(400 μAcm^{-2})		supercapacitor	1M Na_2SO_4	34-36 F g^{-1}		[83]
MnO_2	GP-coated<br>polyester fiber	20 mM $Mn(NO_3)_2$<br>+ 100 mM $NaNO_3$	ED	constant current<br>(100 $\mu A\ cm^{-2}$)		supercapacitor	0.5 M Na_2SO_4	315 F g^{-1}@2 mV s^{-1}	95% after 5000 cycles	[84]
GP/MnO_2 (Nanowall morphology)	GO-modified<br>GCE	0.05 M $Mn(OAc)_2$<br>+ 0.1 M Na_2SO_4	ED	potential cycling<br>(0– –1.5 V vs. Ag/AgCl)<br>(GO was cathodically<br>reduced to GP)		supercapacitor	1 M Na_2SO_4	122 F g^{-1}@10 mV s^{-1}		[85]
Au-MnO_2	GO-coated SS	0.1 M Na_2SO_4<br>+ 0.01 mM $AuCl_3$<br>+ 10 mM $Mn(OAc)_2$	ED	potential cycling<br>(0– 1.2 V Ag/AgCl)<br>(Au was deposited<br>cathodically,<br>GO was reduced to GP)		supercapacitor<br><br>biosensor	0.5 M NaOH<br><br>0.1M PBS<br>(pH 7)	575 F g^{-1}@2.5 Ag^{-1}<br><br>*sensitivity:*<br>35.6 $\mu A\ \mu M^{-1}\ cm^{-2}$<br>for epinephrine	 <br>*LOD:* 24 nM	[86]
MnO_2/GP	Ti	0.04 M $MnSO_4$<br>+ 50 mg L^{-1} GP	ED	constant current<br>(0.8 mA cm^{-2})<br>under supergravity		supercapacitor	6 M KOH	595.7 F g^{-1}@0.5 A g^{-1}	stable after 1000 cycles	[62]
RGO/MnO_2	Ni foam	0.07 M $Mn(OAc)_2$<br>+ 0.07 M Na_2SO_4	ED	constant potential<br>(0.6 V vs. Ag/AgCl)	deposited RGO<br>at –1.4 V<br>vs. Ag/AgCl	supercapacitor	1 M Na_2SO_4	347 F g^{-1}@1 A g^{-1}<br>175 F g^{-1}@20 A g^{-1}	93.1% after 2500 cycles	[88]
$Na_{0.11}MnO_2$/3D GP	Ni foam coated<br>with 3D GP	0.1 M $Mn(OAc)_2$<br>+ 1 M Na_2SO_4	ED	constant potential<br>(0.75 V vs. SCE)		Zn-MnO_2 battery	2 M $ZnSO_4$<br>/ 0.2 M $MnSO_4$	1240 F g^{-1}@0.2 A g^{-1}	90% after 9000 cycles	[89]
MnO_2/RGO	SS	0.1 M $MnSO_4$<br>+ 0.1 M Na_2SO_4<br>+ 0.5 g L^{-1} GO	ED	pulse current<br>(2 and 0.5 mA cm^{-2})	heat treated<br>at 300°C	supercapacitor	0.5 M Na_2SO_4	693.4 F g^{-1}@0.5 A g^{-1}	79.2% after 5000 cycles	[66]
MnO_2/RGO	SS	0.1 M $MnSO_4$<br>+ 0.1 M Na_2SO_4<br>+ 0.5 g L^{-1} GO	ED	pulse current<br>(2 and –2 mA cm^{-2})	heat treated<br>at 300°C	supercapacitor	1 M Na_2SO_4	284.6 $m^2\ g^{-1}$<br>789.0 F g^{-1}@0.5 A g^{-1}	84.8% after 5000 cycles	[90]

(continued on next page)

Table S1. (continued)

Synthesis						Application				Ref.
Deposit on substrate	Substrate	Bath composition<br>for ED (or EPD)	Strategy	Procedure<br>for ED (or EPD)	Post-treatment	Area	Test electrolyte	Representative performances		
MnO_2	GO-Ni foam	1.5 g L^{-1} MnO_2	EPD	constant voltage<br>(2 – 4 V)		ORR	0.1 M KOH	*kinetic current density:*<br>1.1 mA cm^{-2}		[91]
Carbon nanotubes										
MnO_2 nanowires	CNT paper	0.1 M Na_2SO_4<br>+ 0.1 M $Mn(OAc)_2$	ED	potential cycling<br>(0.6 – 0.3 V vs. SCE)		supercapacitor	0.1 M Na_2SO_4	168 F g^{-1}@77 mA g^{-1}	88% after 3000 cycles	[92]
MnO_2	CNT film	0.1 M $Mn(OAc)_2$	ED	constant potential<br>(0.7 V vs. Ag/AgCl)		Zn-MnO_2 battery	2 M $ZnSO_4$<br>+ 0.2 M $MnSO_4$	312 mAh g^{-1}<br>@0.2 mA cm^{-2}	100% after 1000 cycles	[93]
MnO_2 nanofibers<br>/CNT	SS	3.3 g L^{-1} MnO_2<br>+ 0.1 g L^{-1} SA<br>+ 0.1 g L^{-1} CNTs	EPD	constant voltage<br>(15 V)		supercapacitor	0.1 M Na_2SO_4	<200 F g^{-1}		[94]
MnO_2/CNT	SS	9 g L^{-1} MnO_2<br>+ 15 mg L^{-1} dopamine<br>+ 0.6 g L^{-1} CNTs	EPD	constant voltage<br>(20 V)		supercapacitor	0.5 M Na_2SO_4	650 F g^{-1}@2 mV s^{-1}		[96]
MnO_2/CNT/GP	Ni foil	80 mL isopropyl alcohol<br>+ 0.1 g MnO_2/CNT/GP<br>+ 2 mL HCl (37%)	EPD	constant voltage<br>(50 V)		supercapacitor	0.1 M Na_2SO_4	481 F g^{-1}@5 mV s^{-1}	83.3% after cycles	[72]
MnO_2/CNT	SS	1 g L^{-1} MnO_2 + 0.2 g L^{-1}<br>calconcarboxylic acid<br>+ 0.13 g L^{-1} CNTs in<br>ethanol	EPD	constant voltage<br>(40 V)		supercapacitor	0.5 M Na_2SO_4	290 F g^{-1} (44 mF cm^{-2})<br>@ 2 mV s^{-1}		[71]
MnO_2/GP-patched<br>CNT	CNT paper	0.6 M $MnSO_4$<br>+ 0.8 M H_2SO_4<br>at different temperatures	ED	constant current	soaked<br>MnO_2/CNT paper<br>in a 50 μg L^{-1} GP<br>solution	supercapacitor	1 M Na_2SO_4	190.8 F g^{-1}	92.8 % after 800 cycles	[97]
MnO_2/CB/C	Ni plate	1% SA + MnO_2 + CB<br>(MnO_2:CB:SA=8:1:1)	EPD	constant voltage<br>(+20 V)	annealed at 700°C<br>for carbonization<br>of SA	supercapacitor	0.5 M Na_2SO_4	30 mF cm^{-2}<br>@10 mV s^{-1}	98% after 1500 cycles	[98]
MnO_2/CNT	SS	0.5 g L^{-1} humic acid<br>+ 4 g L^{-1} MnO_2<br>+ 0.5 g L^{-1} CNTs<br>in ethanol/water	EPD	anodic, 100 V		supercapacitor		202 F g^{-1} (0.84 F cm^{-2})<br>@2 mV s^{-1}		[99]
MnO_2 with conducting polymers										
MnO_2/PEDOT<br>coaxial nanowires	Au-sputtered<br>AAO<br>membrane	0.01 M $Mn(OAc)_2$<br>+ 0.08 M PEDOT<br>+ 0.1 M $LiClO_4$<br>+ 0.14 M SDS	ED	constant potential<br>(0.7 V vs. Ag/AgCl)		supercapacitor	1 M $LiClO_4$	270 F g^{-1}		[100]

(continued on next page)

Table S1. (continued)

Synthesis						Application				Ref.
Deposit on substrate	Substrate	Bath composition<br>for ED (or EPD)	Strategy	Procedure<br>for ED (or EPD)	Post-treatment	Area	Test electrolyte	Representative performances		
PANI-MnO_2	CC	0.8 mL aniline monomer<br>+ 30 mL of H_2SO_4<br>+ 507 mg $MnSO_4$	ED	constant potential<br>(0.9 V vs. Ag/AgCl)		Zn-MnO_2 battery	2 M $ZnSO_4$<br>+ 0.2 M $MnSO_4$	285.3 mAh g^{-1}<br>@0.5 A g^{-1}	stable after 1200 cycles	[101]
MnO_2@CPPy<br>CPPy: paertially carbonized PPy	CC	0.1 M $Mn(OAc)_2$	ED	constant current<br>(10 mA cm^{-2})	soaked in 5 wt% $FeCl_3$ ethanol<br>then, annealed at 300°C	supercapacitor	1 M KOH	317 F g^{-1}@10 mV s^{-1}	96% after 5000 cycles	[102]
PPy/MnO_2	CC	0.5 M $Mn(OAc)_2$<br>+ 0.1 M Na_2SO_4	ED	constant potential<br>(0.92 V vs SCE)	polymerized at 0.8 V<br>in 0.1 M $NaClO_4$<br>+ 0.2% pyrrole solution	supercapacitor	1 M Na_2SO_4	325 F g^{-1}@0.2 A g^{-1}	96% after 1000 cycles	[103]
Ternary composites										
PPy/GO/MnO_x	Ni foam	0.1 M pyrrole<br>+ 1 g L^{-1} GO<br>+ 0.1 M of NapTS<br>+ 0.1 M of $MnSO_4$	ED	constant potential<br>(0.8 V vs. Ag/AgCl)		supercapacitor	1 M Na_2SO_4		96.58%	[109]
RGO/MnO_2/PANI	RGO paper	0.1 M $Mn(OAc)_2$<br>+ 0.1 M Na_2SO_4	ED	constant potential<br>(0.8 V vs. Ag/AgCl)	dipped RGO/MnO_2<br>in aniline + CSA + APS solution	supercapacitor	1.0 M Na_2SO_4	638.5 F g^{-1}@1.0 A g^{-1}	85% after 10,000 cycles	[105]
PANI-MnO_2-GP	FTO	2 mg GP<br>+ 0.1 M aniline<br>+ 0.1 M Na_2SO_4<br>+ 0.075 M $MnSO_4$<br>+ 1 M H_2SO_4	ED	constant potential<br>(1.2 V vs SCE)		supercapacitor	0.5 M H_2SO_4	243.0 F g^{-1}@5 mV s^{-1}	70% after 450 cycles	[110]
Other composites										
MnO_2@Mxene	CC	0.1 M $Mn(OAc)_2$<br>+ 0.01 M Na_2SO_4	ED	constant current<br>(1 mA cm^{-2})	immersed in Mxene dispersion	Zn-MnO_2 battery	2 M $ZnSO_4$<br>+ 0.2 M $MnSO_4$	517.0 mAh g^{-1}<br>@0.1 A g^{-1}	51.4% after 800 cycles<br>@1 A g^{-1}	[108]

[a] ED: electrodeposition; EPD: electrophoretic deposition; GP: graphene; RGO: reduced graphene oxide; OAc: CH_3COO^-; GCE: glassy carbon electrode; SS: stainless steel; CB: carbon black; SA: sodium alginate; AAO: anodic aluminum oxide; SDS: sodium dodecyl sulfate.

[b] Specific capacitance and stability for supercapacitor application; Sensity and low detection limit for sensor application; Specific capacity and stability for battery application; Current density for catalyst application.

Table S2. Various synthetic procedures of MnO_2-based nanocomposites as OER/ORR bifunctional catalysts via electrodeposition reported in the literature [a]

Deposit on substrate	Substrate	Bath composition for ED	Procedure for ED	Post-treatment	Application	Ref.
α-Mn_2O_3	GC	0.1 M $Mn(OAc)_2$ + 0.1 M Na_2SO_4	constant potential (0.4 V vs SCE)	annealed at 480°C in air	OER/ORR	[6]
MnO_2 nanoneedles	CC	0.1 M $Mn(OAc)_2$ + 0.1 M Na_2SO_4 + SDS	constant potential (<1.6 V vs Hg/HgO/20% KOH)		OER/ORR (ZAB)	[151]
separated MnO_2-NiFe	Ni foam	0.1 M $Mn(OAc)_2$ + 0.1 M Na_2SO_4	constant potential (0.6 V vs. SCE)		OER/ORR (ZAB)	[152]
separated MnO_2-Co_3O_4	Ni foam	Agar Mn hydrogel (0.05 M $Mn(NO_3)_2$ + 0.075 M $NaNO_3$)	constant potential (1.0 V vs Ag/AgCl)		OER/ORR (ZAB)	[154]
MnO_2/CNT	CFP coated with CNTs	0.1 M $Mn(OAc)_2$ + 0.1 M Na_2SO_4	constant current (5 mA cm^{-2})		OER/ORR (LAB)	[153]
Fe_2O_3@C@MnO_2	Ni foam coated with Fe_2O_3@C	0.1 M $Mn(OAc)_2$ + 0.1 M Na_2SO_4	constant current (1.5 mA cm^{-2})	annealed at 230°C in air	OER/ORR (LAB)	[155]
sponge-like *ε*-MnO_2 nanostructure	Ni foam	0.1 M $Mn(OAc)_2$ + 0.1 M Na_2SO_4	constant current (5.0 mA cm^{-2})	annealed at 150～350°C in air	OER/ORR (LAB)	[156]
Mn	SS felt	0.1 M $Mn(OAc)_2$ + 0.1 M Na_2SO_4	constant current (0.25 mA cm^{-2})	hydrothermally treated at 100, 125, 500°C	OER/ORR (LAB)	[157]
MnO_2-Co_3O_4@CC	CC coated with Co_3O_4	0.01 M $Mn(OAc)_2$ + 0.02 M NH_4OAc	constant current (0.4 mA)	annealed 400°C in air	OER/ORR (LAB)	[158]

[a] ZAB: zinc-air battery; LAB: lithium-air batte
